# Relevance of Trypanothione Reductase Inhibitors on *Trypanosoma cruzi* Infection: A Systematic Review, Meta-Analysis, and In Silico Integrated Approach

**DOI:** 10.1155/2018/8676578

**Published:** 2018-10-24

**Authors:** Andréa Aparecida Santos Mendonça, Camila Morais Coelho, Marcia Paranho Veloso, Ivo Santana Caldas, Reggiani Vilela Gonçalves, Antônio Lucio Teixeira, Aline Silva de Miranda, Rômulo Dias Novaes

**Affiliations:** ^1^Institute of Biomedical Sciences, Federal University of Alfenas, Alfenas, 37130-001 Minas Gerais, Brazil; ^2^Department of Structural Biology, Federal University of Alfenas, Alfenas, 37130-001 Minas Gerais, Brazil; ^3^Faculty of Pharmaceutical Sciences, Federal University of Alfenas, Alfenas, 37130-001 Minas Gerais, Brazil; ^4^Department of Pathology and Parasitology, Federal University of Alfenas, Alfenas, 37130-001 Minas Gerais, Brazil; ^5^Department of Animal Biology, Federal University of Viçosa, Viçosa, 36570-000 Minas Gerais, Brazil; ^6^Interdisciplinary Laboratory of Medical Investigation, School of Medicine, Federal University of Minas Gerais, Belo Horizonte, 30130-100 Minas Gerais, Brazil; ^7^Institute of Biological Sciences, Department of Morphology, Federal University of Minas Gerais, Belo Horizonte, 30130-100 Minas Gerais, Brazil

## Abstract

Due to the rudimentary antioxidant defenses in *Trypanosoma cruzi*, disruptors of redox balance are promising candidates for new antitrypanosomal drugs. We developed an integrated model based on systematic review, meta-analyses, and molecular modeling to evaluate the effect of trypanothione reductase (TR) inhibitors in *T. cruzi* infections. Our findings indicated that the TR inhibitors analyzed were effective in reducing parasitemia and mortality due to *Trypanosoma cruzi* infection in animal models. The most investigated drugs (clomipramine and thioridazine) showed no beneficial effects on the occurrence of infection-related electrocardiographic abnormalities or the affinity and density of cardiac *β*-adrenergic receptors. The affinity between the tested ligands and the active site of TR was confirmed by molecular docking. However, the molecular affinity score was unable to explain TR inhibition and *T. cruzi* death *in vitro* or the antiparasitic potential of these drugs when tested in preclinical models of *T. cruzi* infection. The divergence of in silico, *in vitro*, and *in vivo* findings indicated that the anti-*T. cruzi* effects of the analyzed drugs were not restricted to TR inhibition. As *in vivo* studies on TR inhibitors are still scarce and exhibit methodological limitations, mechanistic and highly controlled studies are required to improve the quality of evidence.

## 1. Introduction

Chagas disease is a neglected tropical disease caused by the protozoan parasite *Trypanosoma cruzi*. Recent estimates indicate that 6-7 million people are infected by this parasite worldwide, while at least 120 million people are at risk of infection [1, 2]. Chagas disease is associated with poverty and is endemic in Central and South American countries. Due to the migratory flow of infected people, cases of infection registered in nonendemic areas are on the rise, especially in North America (over 300,167 cases) and Europe (almost 181,181 cases) [1, 2]. While vector-borne transmission, intake of contaminated foods, and congenital transmission are the main forms of *T. cruzi* infection in endemic countries [3–5], autochthonous iatrogenic cases secondary to blood transfusion, organ transplant from infected donors, and congenital transmission are the most frequent infection pathways in nonendemic regions [3, 5].

Chagas disease is a life-threatening illness associated with at least 50,000 deaths/year worldwide, especially due to sudden cardiac death (60%), heart failure (25%), and stroke (15%) [6]. Although the course of infection includes neural (autonomic neuropathy) and digestive disorders (mega syndromes), chronic Chagas cardiomyopathy (CCC) is the most severe and incapacitating clinical form of the disease [3, 7]. Approximately 30% of the infected patients progress to CCC which manifests as diffuse heart fibrosis and hypertrophy, complex electrical abnormalities and arrhythmias, thromboembolic events, and congestive heart failure [3, 8]. Chronic Chagas cardiomyopathy is the most common cause of nonischemic cardiomyopathy in South America [9] and the third most common cause of heart transplantation in Brazil [10]. CCC is also associated with a higher mortality hazard ratio (2.48) than noninfectious cardiomyopathies [9, 11].

Due to limited effectiveness of the strategies to control parasite transmission (i.e., vector control, screening of infected pregnant women, and blood and organ banks) and infection treatment, Chagas disease incurs US $7.19 billion per year in healthcare costs, and more than 10% of that amount comes from nonendemic countries such as the USA and Canada [12]. Nifurtimox- and benznidazole-based chemotherapy (developed more than 40 years ago) remains the main strategy for the etiological treatment of Chagas disease [13, 15]. Although these drugs present acceptable effectiveness in acute infections (an approximately 60% cure rate), they are highly toxic and achieve low cure rates (10%–20%) in chronic infections. As nifurtimox is no longer used in most Central and South American countries due to excessive side effects (i.e., hypersensitivity reactions, polyneuritis, toxic hepatitis, bone marrow depression, immunosuppression, and cancer), benznidazole is often the only drug available [13, 14]. As progress in drug development has been very limited in recent decades, new and less toxic antitrypanosomal treatments are urgently needed [7, 16].

Considering that most neglected tropical diseases are not included in the research and development platforms of pharmaceutical industries [14, 17], the prospect of new drugs for the treatment of Chagas diseases is not promising. Thus, drug repositioning by identifying commercially available products with anti-*T. cruzi* potential [18] provides a strong rationale and viable screening option [19]. From the characterization of a rudimentary metabolic pathway associated with antioxidant defenses in trypanosomatids [20, 21], disruptors of redox balance are proposed as candidates for new antitrypanosomal drugs [16, 20, 22]. There is evidence that the enzyme trypanothione reductase (TR) plays a pivotal role in maintaining the functional integrity of antioxidant systems in trypanosomatids. Accordingly, inhibition of this FAD-cystine-oxidoreductase is effective in increasing *T. cruzi* susceptibility to oxidative stress, which together with the immune system integrates the host defenses against parasitic infections [23, 24]. As TR is not expressed in vertebrate hosts, this enzyme represents a potentially useful molecular target for rational drug design [16, 20, 25].

Several anti-inflammatory, antineoplastic, antidepressant, anxiolytic, and antipsychotic drugs are TR inhibitors [16, 26], and their specific effects are potentially mediated by interaction of these drugs with active sites on TR, especially the FAD- and NADPH-binding domains [22]. As current knowledge of enzyme inhibition and antitrypanosomal effects is based on in silico [16, 27] and *in vitro* [28, 29] systems, it is unknown if and to what extent these effects can be reproduced *in vivo*. In addition to being fragmented, the current evidence is based on few research initiatives, and the specific inhibitors used and their effectiveness at modifying the pathogenesis of *T. cruzi* infection are still obscure.

In the current manuscript, we systematically review the *in vivo* preclinical evidence on the relevance of TR inhibitors in Chagas disease. In addition to exploring the main characteristics of the experimental models, parasite strains, and protocols of treatment, meta-analyses were used to calculate the effect sizes and direction of parasitological, biochemical, and electrophysiological outcomes. From an integrated in silico approach, we also investigated if and to what extent the effect size obtained for each drug could be explained by variations in chemical structures, molecular interactions, and the rate of TR inhibition. The methodological quality of each study identified was also evaluated, and the main sources of bias that undermine the quality of evidence were pointed out.

## 2. Methodology

### 2.1. Search Strategy

The search strategy was based on two steps according to Pereira et al. [1] and based on PRISMA (Preferred Reporting Items for Systematic Reviews and Meta-Analyses) statement [30]. A direct search was carried out in three comprehensive electronic databases: PubMed/MEDLINE, Web of Science, and Scopus. A secondary search was based on screening of the reference list of all relevant studies identified in the direct search.

Structured search filters were developed for each database (Table S1). The search filters were initially constructed by considering standardized descriptors (Medical Subject Headings (MeSH)) extracted from PubMed thesaurus. All descriptors were combined in a complete three-level search strategy based on (i) *in vivo* preclinical models, (ii) disease (American trypanosomiasis), and (iii) therapeutic intervention (TR inhibitors). Standardized descriptors were defined by the MeSH algorithm, and non-MeSH descriptors were characterized by the TIAB algorithm, which was also used to recover recently published but nonindexed studies (*in process*). A previously published and optimized animal filter was applied in the PubMed search interface [31]. The same search filters used for disease and intervention were adapted for Web of Science and Scopus. An animal filter was created for Web of Science considering the animal models identified in all studies recovered from the PubMed/MEDLINE database. Scopus' own animal filter (keyword—animals [limit to]) was used in this database. No chronological or language limits were applied in our search strategy. All relevant studies published until September 30, 2017 (updated search date), were recovered and included in the systematic review and meta-analysis.

### 2.2. Record Screening and Eligibility

All research records recovered in the database search were analyzed, and duplicates were removed considering the authors, title, journal, and year of publication. After title and abstract screening, all potentially relevant studies were evaluated in full text for eligibility according to specific inclusion and exclusion criteria. Only studies investigating the relevance of TR inhibitors to *in vivo* preclinical models of Chagas disease were included in the review. The exclusion criteria were (i) no full-text available, (ii) secondary studies (i.e., editorials, commentaries, and letters to the editor), (iii) observational and epidemiological reports, (iv) studies without control groups, and (v) studies exclusively investigating *in vitro* and human systems. Literature reviews were considered only when original data were additionally reported. Eligibility was independently analyzed by the researchers A.A.S.M, R.D.N, and R.V.G. Disagreements were resolved by consensus. To enhance the comprehensiveness of the research strategy, the reference lists of all relevant papers identified from database searches were screened for additional studies.

### 2.3. Data Extraction and Synthesis

Data extraction was based on basic methodological requirements for preclinical studies, as previously described by Pereira et al. [1]. Considering a detailed characterization of all relevant studies identified, essential data grouped into four descriptive levels were extracted and summarized as follows: (i) publication characteristics: authors, publication year, and countries; (ii) experimental design (animal model): species, lineage, sex, age, and weight; (iii) experimental design (disease model): parasite species and strain, inoculum size, route of parasite administration, and duration of infection; and (iv) main research outcomes (i.e., parasitemia, parasitic load, immunological markers, histopathological findings, and mortality).

Quantitative data on key outcome measures commonly investigated in preclinical *in vivo* parasitological studies on anti-*T. cruzi* chemotherapy were extracted. Mean and standard deviation (mean ± SD) were directly extracted when available in tables or text. When these data were reported in graphics, an image analysis software program (Image-Pro Plus 4.5, Media Cybernetics, MD, USA) calibrated to each image was used to extract these values.

### 2.4. Molecular Docking

Molecular docking analysis was performed in Schrödinger software suite Maestro (Schrödinger, New York, USA) version 10.2.010 using the crystal structure of *Trypanosoma cruzi* TR (PDB code: 1BZL) with two chains. For ligand preparation, the LigPrep program was used with an OPLS-3 force field and ionization state for pH 7.0 ± 2.0 (Schrödinger, New York, USA). To the ligands containing metals, the bonds between the metal and the atoms were turned into zero-order bonds, and the formal charges on the atoms were accordingly adjusted to +2 on the manganese atom and to −1 on the oxygen atoms. Protein structure preparation was performed by using the Protein Preparation Wizard program, with hydrogen bonding network optimization at pH 7.0 and minimization performed using the OPLS-3 force field in the MacroModel module (Schrödinger, New York, USA).

For docking analysis, the Induced Fit Docking (IFD) protocol was used, which predicted the protein structure and docking, refined the compounds by using the Prime program, and provided the score by using the Glide program, considering the protein and the ligand to be flexible [32]. The grid box area was defined as 20 × 20 × 20 Å in the active site region. The force field used was OPLS-3. The final ligand protein complexes were visualized using the Maestro interface, and figures were generated using its graphical interface.

### 2.5. Methodological Bias

Reporting bias was analyzed according to Pereira et al. [1] and based on methodological requirements described in the Animal Research: Reporting of *In Vivo* Experiments guidelines [33]. This strategy requires the complete screening of all manuscript sections (abstract to acknowledgements and funding) to evaluate the completeness of scientific reports on animal studies. The screening strategy was based on short descriptions of essential characteristics such as baseline measurements, sample size, animal allocation, randomization, experimental concealment, statistical methods, ethical statement, and generalizability. A table summarizing all relevant and applicable aspects was constructed considering the specificity and aims of the systematic review. The individual adherence to bias criteria and the overall mean adherence were expressed as absolute and relative values [1].

### 2.6. Statistical Analysis

Taking into account potential heterogeneity in the studies identified, we used a statistical model based on random-effects weighted mean difference meta-analysis, in which some heterogeneity and sampling error are admitted to calculate a summary estimate of effect size and its 95% confidence intervals [34]. For this model, standard error (SE) was converted to standard deviation using the formula $SD=SEx\sqrt{n},$ where *n* is the number of animals used in each experimental group. The variability of each outcome assessed was presented as the heterogeneity statistic (*I* ^2^) [35]. For dichotomous (survival/mortality and electrocardiographic normality/abnormality) and continuous variables, the risk ratio (RR) and standardized mean difference (SMD) were used as estimates of effect, respectively. Where outcomes were repeatedly measured, we established two time points of infection (acute phase and chronic phase) at which the mean result was calculated for each phase. From the relative similarity among the experimental models, subgroup analyses based on variables such as mouse lineage, weight, and parasite strain were not applicable to exploring heterogeneity. The sex of animals was not admitted, because there is no evidence of a sex-dependent pharmacological response for the drugs investigated or any anti-*T. cruzi* drug. Furthermore, age-based heterogeneity was not taken into consideration as this variable was underreported. When appropriate, subgroup analysis was based on methodological (specific drugs and phase of infection) rather than biological characteristics to try to explain possible causes of heterogeneity. A subgroup was defined as a group containing a minimum of two studies [36].

## 3. Results

### 3.1. Publication Characteristics

**Figure 1:**
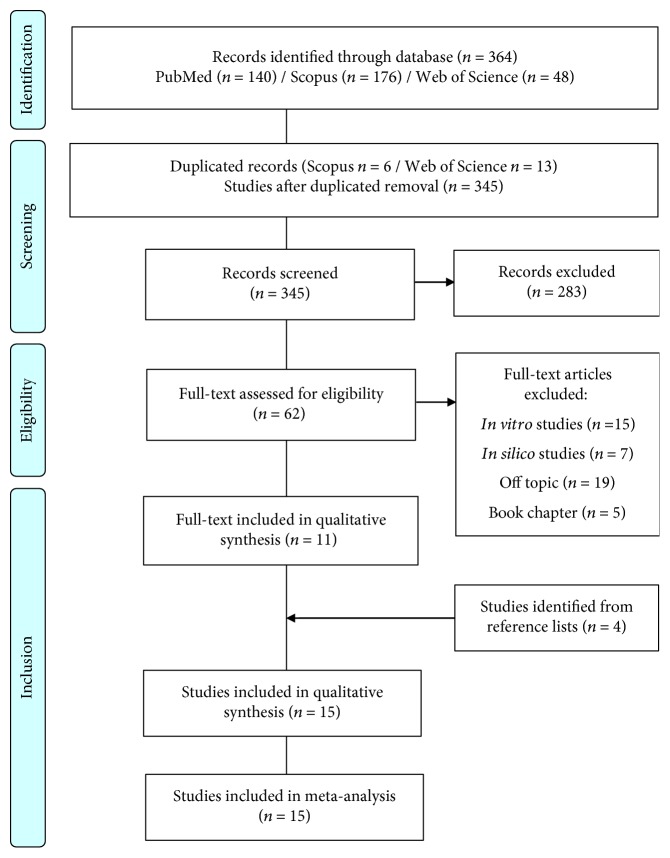
Flow diagram with the search results obtained in the systematic review. Based on PRISMA statement “Preferred Reporting Items for Systematic Reviews and Meta-Analyses” (http://www.prisma-statement.org).

From our search strategy, 15 relevant studies (11 identified in electronic databases and 4 recovered in the secondary search) were included in the systematic review (Figure 1). Most of these studies originated in South America countries, especially Argentina (80%, *n* = 12), followed by European countries (20%, *n* = 3).

### 3.2. Reporting Bias

**Figure 2:**
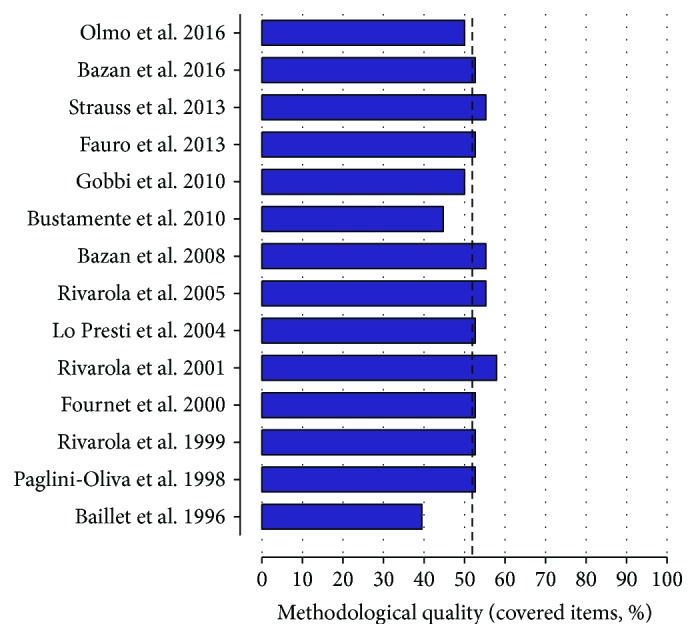
Analysis of methodological bias (reporting quality) for each study included in the review. The dotted line indicates the mean quality score (%). Drugs investigated: Olmo et al. 2016 a, b and c (compound 2, compound 3, and compound 5); Lo Presti et al. [26], Bustamante et al. 2010, Lo Presti et al. [37], Rivarola et al. [41], and Paglini-Oliva et al. 1998 (thioridazine); Fournet el at. 2000 a and b (daphnoline and cepharanthine); Baillet et al. [125] (2-aminodiphenylsulfide); and Bazan et al. 2016, Strauss et al. [40], Fauro et al. [39], Gobbi et al. [42], Bazan et al. 2008, Rivarola et al. [38], and Rivarola et al. [83] (clomipramine). Detailed bias analysis stratified by domains and items evaluated is presented in Table S7.

Detailed results of bias analysis are shown in Table S2 and Figure 2. The original studies adhered to a mean of 52.3 ± 5.6 bias items (Figure 2). No study reported experimental blinding, baseline procedures and measures, housing of experimental animals (type of facility or housing), sample size calculation, welfare-related assessments and interventions, order in which the groups were treated and assessed, adequacy of the statistical approach, animal or data exclusion, and study limitations (sources of intrinsic and extrinsic bias). The rationale for the choice of administration route and details of animal allocation (7.9%, *n* = 1) and the rational basis for dosage definition and age of the animals were also underreported (14.2%, *n* = 2). Fifty-seven percent of studies presented clear objectives and ethical approval. Information on parasitemia, mortality, parasite strain, and mouse weight was cited in 11 studies (78.6%). Sex of the animals, experimental conditions, number of animals, relevance to human biology, statistical methods (85.7%, *n* = 12), and route of administration (92.8%, *n* = 13) were consistently reported.

### 3.3. Characteristics of the Animal Models

All studies used mice as the animal model. Swiss mice were the main lineage used (73.3%, *n* = 11), and only two studies (13.33%) used BALB/c mice. Forty percent of studies (*n* = 6) used male animals, 13.3% (*n* = 2) used female animals, and 26.6% (*n* = 4) used animals of both sexes. Three studies (20%) did not specify the sex of the animals. The animals' age was neglected in most studies (86.6%, *n* = 13), and the animals' weight ranged from 20 to 30 g. Tulahuen (53.3%, *n* = 8), SGO-Z12 (13.33%, *n* = 2), or both (13.33%, *n* = 2) *T. cruzi* strains were used to induce infection. Parasite strain was not mentioned by three studies (20%) (Table S3).

### 3.4. Characteristics of the Treatments

As shown in Table S3, clomipramine (46.6%, *n* = 7), thioridazine (33.3%, *n* = 5), tetraamine-based compounds (6.6%, *n* = 1), bisbenzylisoquinoline alkaloids (6.6%, *n* = 1), and metallodrugs (6.6%, *n* = 1) were the drugs tested against *T. cruzi* infection. The doses administered ranged from 4 mg/kg to 80 mg/kg. The administration routes were intraperitoneal (46.6%, *n* = 7), oral (40%, *n* = 6), or both (6.6%, *n* = 1). The treatment period ranged from 1 hour to 90 days.

### 3.5. Parasitological Outcomes and Mortality

**Figure 3:**
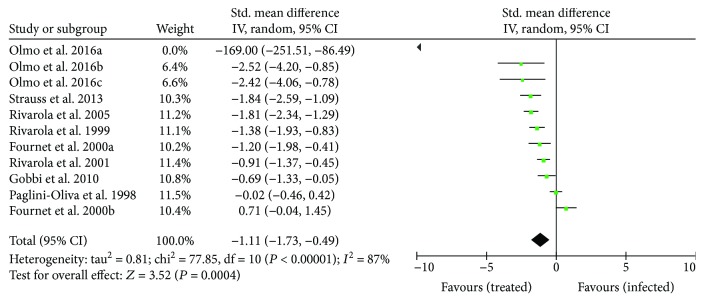
Forest plot obtained from meta-analysis comparing the mean difference of parasitemia in infected mice treated with trypanothione reductase inhibitors and those untreated (control). Drugs investigated: Olmo et al. [43] b and c (compound 2, compound 3, and compound 5); Fournet el at. [95] and b (daphnoline and cepharanthine); Rivarola et al. [41] and Paglini-Oliva et al. 1998 (thioridazine); and Rivarola et al. [38], Strauss et al. [40], Rivarola et al. 2000, and Gobbi et al. [42] (clomipramine).

The results of parasitemia and mortality for each study are detailed in Table S4. In general, TR inhibitors were effective in controlling parasitemia and mortality. When the studies were grouped by drug and by dose investigated, no heterogeneity (*I* ^2^ = 0) or significant differences were detected among subgroups (*P* = 0.41). Thus, the overall effect of treatment on parasitemia was analyzed for the set of studies reporting these data. Eight of nine studies showed a significant reduction in parasitemia, indicating a consistent effect in favor of treated animals compared with untreated infected mice (SMD: −1.11 [95% CI: −1.73, −0.49], *P* = 0.0004) (Figure 3).

As shown in Table S5, only one study (20%, *n* = 1) objectively reported parasitic load, indicating reduced heart parasitism in animals treated with clomipramine (5 mg/kg). Parasitological cure was investigated in four studies (80%, *n* = 4). The cure rate (CR) was similar in all of them, except for those using cepharanthine and daphnoline (CR = 51% and 84%, respectively, vs. infected untreated mice CR = 17%) and two tetraamine-based metallodrugs (CR = 33.33% and 50%, vs. infected untreated mice CR = 0%).

**Figure 4:**
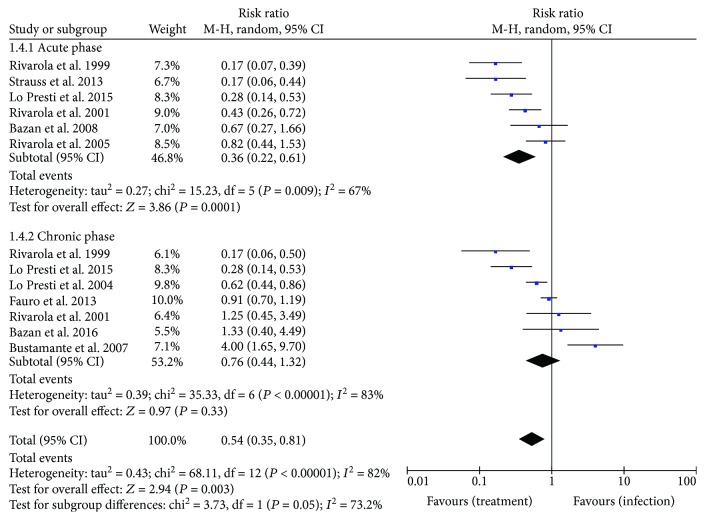
Forest plot obtained from meta-analysis comparing the risk ratio for mortality in infected mice treated with trypanothione reductase inhibitors.

Overall mortality (Figure 4) was significantly reduced in animals treated with TR inhibitors (RR: 0.54 [95% CI: 0.35, 0.81], *P* = 0.003). A favorable effect of treatment on mortality (*P* < 0.05) was observed in the acute phase of infection (RR: 0.36 [95% CI: 0.22, 0.61]). Conversely, these drugs had no impact (*P* > 0.05) on mortality in chronic infections (RR: 0.76 [95% CI: 0.44, 1.32]).

### 3.6. Electrocardiographic Changes and *β*-Adrenergic Receptors

**Figure 5:**
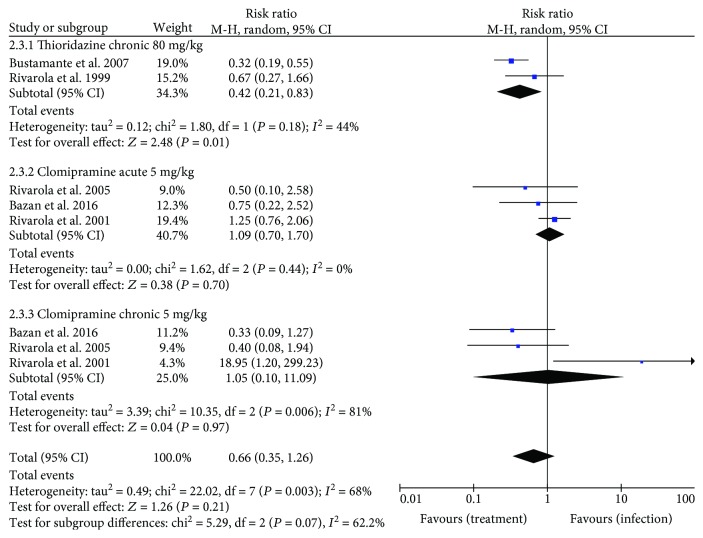
Forest plot obtained from meta-analysis comparing the risk ratio for electrocardiographic abnormalities in infected mice treated with trypanothione reductase inhibitors and those untreated (control). Abnormalities were determined by evidence of arrhythmias and intraventricular block.

Electrocardiographic findings are detailed in Tables S6 and S7. Electrocardiographic and cardiac receptor data were reported in seven studies (46.6%, *n* = 7) investigating clomipramine or thioridazine. The overall effect showed no difference in electrocardiographic abnormalities (i.e., arrhythmias and intraventricular block) comparing treated and untreated infected mice (RR: 0.66 [95% CI: 0.35, 1.26], *P* = 0.21). In subgroup analysis, treatment with thioridazine reduced the occurrence of electrocardiographic abnormalities in chronic infection compared with untreated mice (RR: 0.42 [95% CI: 0.21, 0.83). Conversely, the risk of electrocardiographic disturbances in both phases of infection was similar (*P* > 0.05) in clomipramine-treated and untreated animals (acute phase RR: 1.09 [95% CI: 0.70–1.70] and chronic phase RR: 1.05 [95% CI: 0.10, 11.09]) (Figure 5).

**Figure 6:**
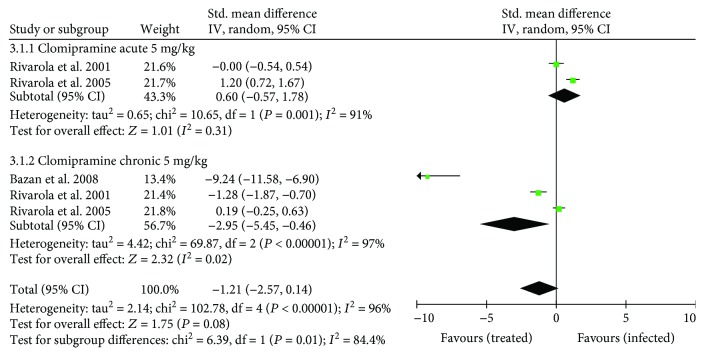
Forest plot obtained from meta-analysis comparing the mean difference of *β*-adrenergic receptor affinity in infected mice treated with trypanothione reductase inhibitors and those untreated (control).

Beta-adrenergic receptor affinity and density are detailed in Table S7. Four studies (57.1%, *n* = 4) analyzed *β*-adrenergic receptors, of which three (42.8%, *n* = 3) using clomipramine were included in the meta-analysis. Considering both phases of infection, *β*-adrenergic affinity presented a similar overall effect when clomipramine-treated and untreated animals were compared (SMD: −1.21 [95% CI: −2.57, 0.14], *P* = 0.08) (Figure 6). Whereas clomipramine-treated animals showed reduced *β*-adrenergic affinity in chronic infection compared with control animals (SMD: −2.95 [95% CI: −5.45, − 0.46], *P* < 0.00001), no significant effect was observed in acute infection (SMD: 0.60 [95% CI: −0.57, 1.78], *P* = 0.31).

**Figure 7:**
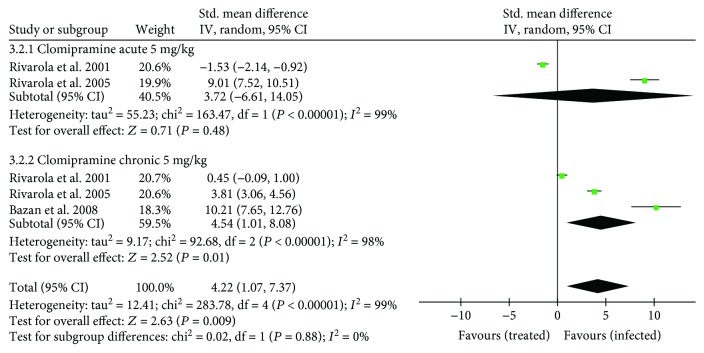
Forest plot obtained from meta-analysis comparing the mean difference of *β*-adrenergic receptor density in infected mice treated with trypanothione reductase inhibitors and those untreated (control).

Considering *β*-adrenergic receptor density, the overall analysis indicated a positive effect in favor of infected untreated compared with clomipramine-treated mice (SMD: 1.15 [95% CI: 0.81, 1.15], *P* < 0.00001) (Figure 7). In subgroup analysis, while untreated animals showed increased *β*-adrenergic density in chronic infection compared with clomipramine-treated animals (SMD: 1.86 [95% CI: 1.43− 2.29], *P* < 0.0001), no significant effect was observed in acute infection (SMD: –0.04 [95% CI: −0.61, 0.52], *P* = 0.88).

### 3.7. Immunological, Histopathological, and Biochemical Findings

Table S8 shows additional morphological, immunological, and biochemical findings. Histopathological data of the heart were reported in 11 studies (73.3%, *n* = 11). In general, TR inhibitors, especially thioridazine and clomipramine, reduced heart inflammation, tissue necrosis, and fibrosis compared with untreated infected mice. Immunoglobulin levels were investigated in 8 studies (53.3%), which generally reported reduced anti-*T. cruzi* antibody levels in animals treated with TR inhibitors. In four studies (26.6%, *n* = 4), antibody levels were similar [37–40], while in two studies (13.3%, *n* = 2), they were greater in treated vs. untreated control groups [41, 42]. Only one study (6.6%, *n* = 1) investigated biochemical parameters of systemic toxicity. In this study [43], while CK-MB, uric acid, and urea serum levels were increased, LDH was reduced (30% and 40%) in metallodrug-treated compared with untreated animals.

### 3.8. In Silico Molecular Interaction

**Figure 8:**
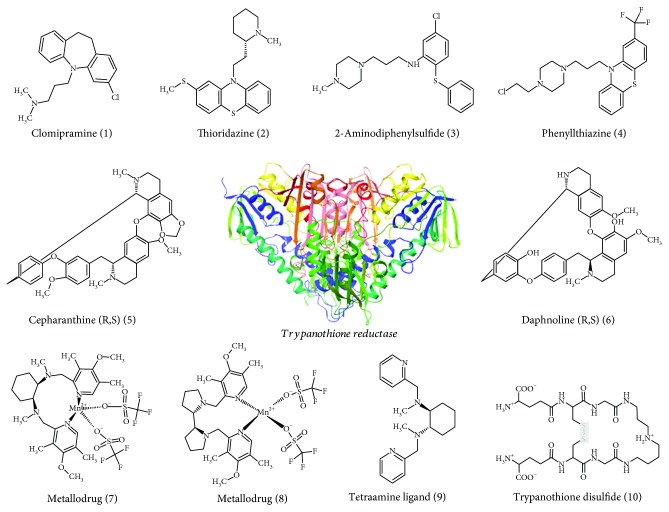
Molecular structures of trypanothione reductase inhibitors (ligands) used in docking studies.

**Figure 9:**
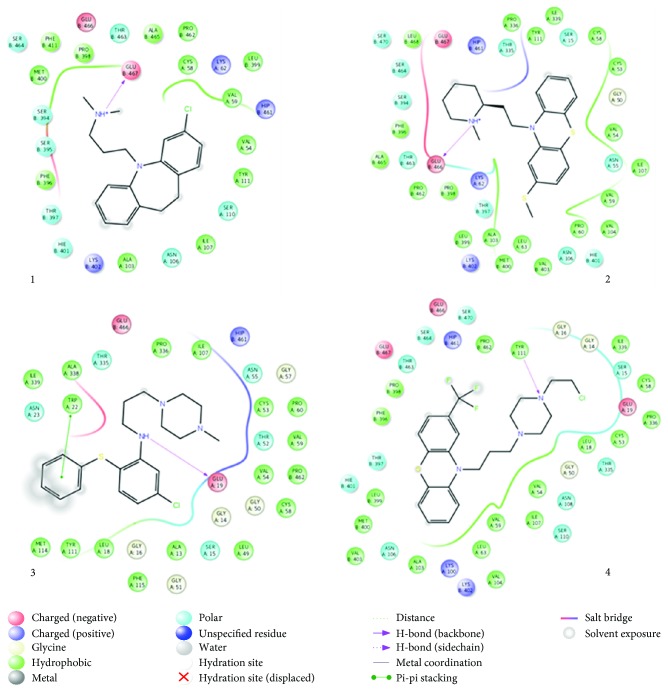
Interactions between amino acids of trypanothione reductase active site and clomipramine (1), thioridazine (2), 2-aminodiphenylsulfide, (3) and phenylthiazine (4).

**Figure 10:**
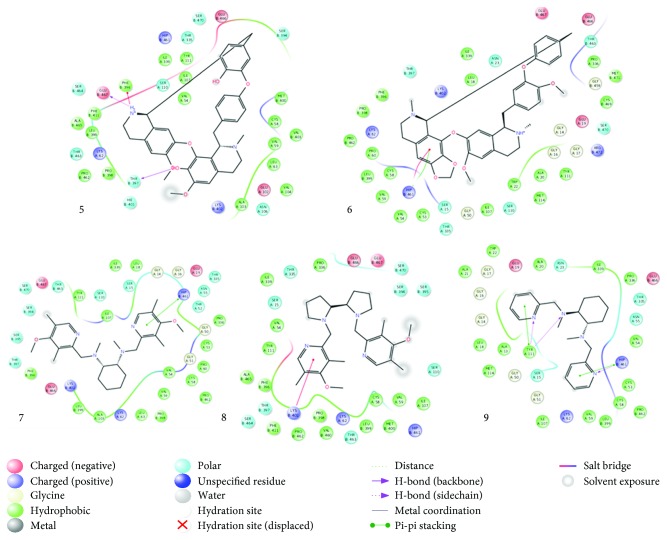
Interactions between amino acids of trypanothione reductase active site and cepharanthine (R,S) (5), daphnoline (R,S) (6), metallodrug (7), metallodrug (8), and tetraamine ligand (9).

Molecular docking of a group of nine different bioactive molecules (ligands) and trypanothione disulfide (substrate) was performed with TR (Figure 8). Values of GlideScore (GScore), the number of interactions by hydrogen bonds (*Hbond*), van der Waals (*good vdW*), *π*-*π* stacking, and cation *π* between the ligands and TR are shown in Table 1. Figures 9 and 10 represent the results of molecular docking in 2D format. Eleven compounds with amino acid residues in the active site of TR showed the types of interactions relevant to these studies.

**Figure 11:**
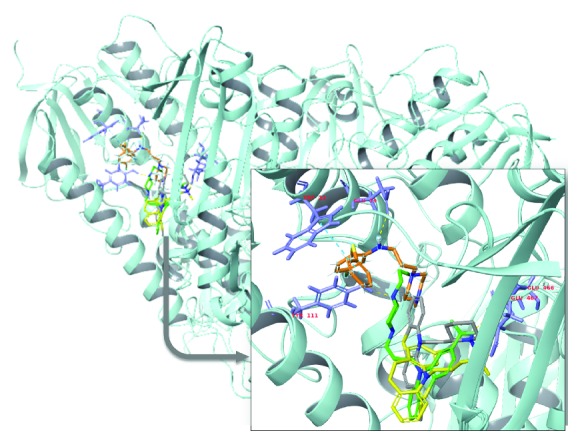
Representation of molecular docking superimposition of clomipramine (1) (yellow carbon), thioridazine (2) (gray carbon), 2-aminodiphenylsulfide (3) (orange carbon), and phenylthiazine (4) (green carbon) with trypanothione reductase active site. The highlighted image shows the interaction of the investigated drugs with the binding site of trypanothione reductase.

**Figure 12 fig12:**
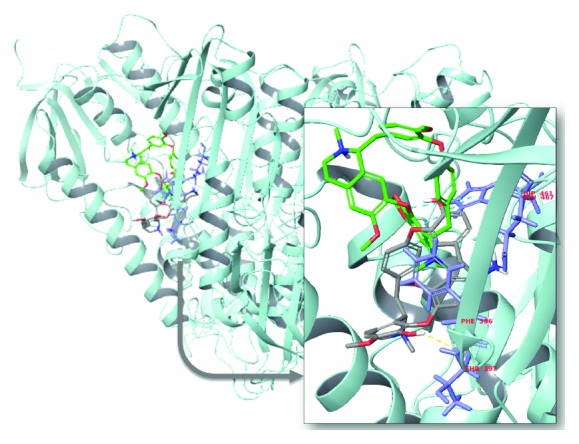
Representation of molecular docking superimposition of cepharanthine (R,S) (5) (green carbon) and daphnoline (R,S) (6) (gray carbon) with the trypanothione reductase active site. The highlighted image shows the interaction of the investigated drugs with the binding site of trypanothione reductase.

**Figure 13:**
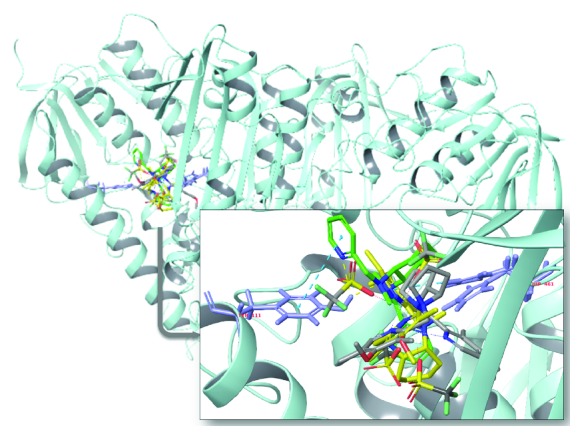
Representation of molecular docking superimposition of metallodrug (7) (gray carbon), metallodrug (8) (yellow carbon), and tetraamine ligand (9) (green carbon) with the trypanothione reductase active site. The highlighted image shows the interaction of the investigated drugs with the binding site of trypanothione reductase.

Figures 11 [Fig fig12]–13 show a superimposition of the molecular docking configurations of the compounds evaluated. The images were divided according to structural similarity. Our results suggested an interaction that could lead to an inhibitory activity of all the compounds towards TR. However, the strength of molecular affinity was not always aligned with the experimental results *in vivo* reported in this work.

## 4. Discussion

The knowledge of *T. cruzi* redox metabolism and the role of oxidative stress in the pathophysiology of Chagas disease have indicated potential targets for antiparasitic chemotherapy [16, 20, 25]. Due to the rudimentary antioxidant defenses, *T. cruzi* is highly susceptible to oxidative and nitrosative events activated during infection of multiples organs, such as the heart [25, 26, 44, 45], skeletal muscles [46], liver [47], and placenta [48]. There is evidence that inflammatory, oxidative, and nitrosative events are coupled processes potentially mediated by proinflammatory cytokines such as TNF-*α*, IFN-*γ*, and IL-1 and IL-6, which stimulates the intense production of reactive species such as oxygen peroxide, superoxide anion, hydroxyl radicals, nitric oxide, and peroxynitrite [45, 49, 50]. In general, decoupling of the electron transport chain in host cells [49], upregulation of endothelial and inducible nitric oxide synthase expression [48, 51], and the respiratory burst in leucocytes recruited in infected tissues [45, 47] are the primary sources of these highly reactive molecules. Reactive oxygen (ROS) and nitrogen (RNS) species are relevant defense molecules against *T. cruzi* infection, and its inhibition (especially NO) has been associated with intense parasite load and severe tissue damage [48, 50]. The blockade of the parasite's antioxidant defenses has been proposed as a relevant strategy to increase host resistance to *T. cruzi* infection [25, 26, 28]. However, due to its interspecific action, host cells are also an important target of ROS and RNS cytotoxicity [45, 47, 49]. Thus, to identify specific molecules involved in the control of redox metabolism in *T. cruzi* may represent a rational and useful strategy to develop antiparasitic drugs with little or no impact on the host's antioxidant defenses. In this sense, TR inhibitors are currently the most promising drugs with direct impact on *T. cruzi* antioxidant defenses.

Most studies investigating TR inhibitors were performed in developing countries, indicating that research efforts are coherently concentrated in South American countries where Chagas disease is endemic [5]. A limited general score of methodological quality was identified for the set of studies. As the analysis of reporting bias was structured from basic requirements to the rational acquisition and interpretation of the results, a limited quality of evidence could be attributed to studies with low methodological scores [52]. Accordingly, a rigorous analysis and interpretation of the available evidence is important, taking into account all critical elements that could hinder construct validity (the degree to which the analytical tool measures what it purports to), internal validity (cause-effect relationships), and external validity (generalizability) of each study, as well as the individual weight associated with meta-analysis effect size [53].

Although individual bias scores were variable, they did not present a temporal influence (year of publication). This finding indicates that reporting bias was systematically reproduced through the research process, independent of notorious advances in the analytical and statistical methods as well as the increasing availability of guidelines and regulatory strategies adopted to stimulate the completeness of the scientific reports in preclinical studies [33]. In all studies included, simple constructs such as experimental blindness, animal allocation and age, sample size calculation, and rational choice of administration route were the main source of bias. Although these elements are important sources of intrinsic bias [54, 55], they also are easily adjustable, and the construction of more rigorous experimental designs aligned with acceptable construct validity can be achieved in future studies.

Despite these methodological limitations, similar elements of experimental design were identified, contributing to the reliability and reproducibility of the results. Parasitemia, mortality, parasite strain, animal number, sex and weight, route of administration, relevance to human biology, and statistical methods were consistently reported. Furthermore, Swiss mice infected by the Tulahuen strain were used as the main animal model. A proper selection of animal species and genetic background is crucial in preclinical investigations of parasitic diseases as these factors are directly related to host resistance and susceptibility to the pathogen [56, 57]. Swiss mice are highly applicable, especially considering that they are highly susceptible to *T. cruzi* infection, and their genotype and phenotype variability resembles the one of the human condition [56, 57]. Swiss mice also recapitulate many features of human disease, especially the immunological profile and histopathological manifestations [58]. From an operational perspective, mice are also good animal models due to ease of handling and housing and the low cost of maintenance. Conversely, the applicability of larger animals as models of Chagas disease, especially dogs and nonhuman primates, is limited by availability, cost, and ethical considerations [1].

Similar strains were also applied to induce *T. cruzi* infection. Careful selection of a parasite strain is essential in preclinical studies, especially considering the variable profiles of infectivity, pathogenicity, and virulence [56, 57, 59]. These elements require good alignment with the outcome measures, which are closely associated with the phase of infection under analysis. Thus, highly pathogenic and virulent parasite strains are realistically applicable only in acute models as the animals often die before developing a chronic infection [60]. Conversely, as parasitological cure and Chagas cardiomyopathy attenuation or reversion are the primary focus of anti-*T. cruzi* chemotherapy, models using pathogenic strains with low virulence are a more rational strategy [60, 61]. Based on these latter models, the chances of instilling morphological, electrical, and mechanical changes typical of Chagas' cardiomyopathy are enhanced [61–63]. Because the parasite strains used were aligned with the phases of interest in Chagas disease, the analyzed studies exhibited an important element of methodological consistence, with a positive reflection on construct validity.

Due to the similarity in experimental designs, analytical methods, and outcome measures, meta-analysis estimates were calculated to evaluate the relevance of TR inhibitors in preclinical treatment of *T. cruzi*. All common and relevant outcomes, such as parasitemia, mortality, electrocardiographic abnormalities, *β*-adrenergic receptor density, and affinity, reported in two or more studies were combined. Taken together, the results of our meta-analyses indicated that these inhibitors are highly effective at attenuating multiple pathological events of experimental Chagas disease. Parasitemia was reduced by TR inhibitors, and no subgroup differences (drugs and doses) were identified. This finding indicated that parasitemia measures were highly consistent and not affected by bias. It is recognized that heterogeneity in meta-analytical studies can be affected by multiple elements of bias, especially those from methodological (experimental design and scientific reporting) or biological sources (genotypic and phenotypic variability) [64, 65]. Thus, controlling all potential sources of bias is essential to ensuring reliable effect sizes in a meta-analysis, with a direct reflection on the quality of evidence [64, 65].

The meta-analysis also indicated that TR inhibitors were effective at reducing overall mortality. However, this effect was determined by benefits limited to acute infections. This finding was coherent with the results of parasitemia and phase of infection. Control of parasitemia is a pivotal objective of antiparasitic chemotherapy, with a direct impact on mortality rates [38, 40, 66]. Parasitemia is integrated in the reproductive cycle of *T. cruzi* and can reflect the parasite's ability to overcome host defenses and propagate the infection [67, 68]. Strategies that control parasite replication are essential to attenuating tissue damage and secondary mortality in Chagas disease [39, 43]. As supported by current evidence, parasitological control is more effective in acute infections with positive repercussions in the chronic phases (i.e., reduction of heart damage) [26, 42, 69]. However, after the parasite spreads and establishes stable reservoirs (amastigote nests) in multiple organs during the chronic infection, antiparasitic treatments exhibit limited effects [70]. The mechanisms associated with this limited efficacy are poorly understood. It is possible that in the late stages of infection, parasites that survive the host's defenses are more resistant to antiparasitic drugs [71, 72]. Low detection limits of circulating parasites by conventional microscopic methods can also be related to the inefficacy of TR inhibitors in chronic infection. As parasitemia decreases to very low or undetectable levels in the chronic phase, the relevance of this parameter to indicate infection is controversial, especially considering that disease progression and development of severe manifestations (i.e., chronic cardiomyopathy) frequently occur in the absence of circulating parasites [73, 74]. Therefore, in chronic infections, parasitic load and specific abnormalities associated with Chagas cardiomyopathy are better parameters to evaluate the efficacy of anti-*T. cruzi* chemotherapy than parasitemia [73].

As cardiac function outcomes were analyzed only in studies testing clomipramine (1) and thioridazine (2), these drugs were combined in a meta-analysis considering subgroup differences (phases of disease and doses) and heterogeneity. All parameters of cardiac function (electrocardiographic abnormalities, *β*-adrenergic receptor density, and affinity) were not influenced by clomipramine and thioridazine treatment. Conversely, an overall effect in favor of *T. cruzi* infection was identified for *β*-adrenergic density. There is no doubt that the heart is highly parasitized and damaged by *T. cruzi* [75, 76]. Structural and electromechanical cardiac damage is the most serious manifestation of Chagas disease and is closely associated with morbidity and mortality, especially in the chronic phase of infection [77–79]. In fact, in all studies included in this review that investigated cardiac function, the main electrocardiographic changes identified in animals infected with *T. cruzi* were atrial and ventricular conduction defects (prolonged PQ and QRS segments) and arrhythmias [80]. These changes were possibly associated with molecular abnormalities in *β*-adrenergic receptors, which are identified as central elements in the pathogenesis of Chagas cardiomyopathy and partially responsible for disturbances in the autonomic regulation of cardiac function [81, 82]. Interestingly, the results of our meta-analysis and the findings reported in the original studies [38, 41, 83] indicated an opposite profile of *β*-adrenergic affinity and density. While receptor affinity was altered by treatment, especially with clomipramine, density was altered by infection. This profile indicates the typical balance between molecular properties of these receptors. There is evidence that damage to *β*-adrenergic receptors is mediated by autoantibodies produced against *T. cruzi* antigens that exhibit molecular mimicry to heart molecules [84, 85]. It is natural that reduction in receptor density from immunological attack is accompanied by a compensatory increase in the affinity of the remaining receptors [25, 86]. This behavior represents an important adaptive and counterregulatory mechanism in the attempt to adjust the cardiac function and to resist organ parasitism [38]. Considering that *T. cruzi* infection is not restricted to the heart, mortality rates are determined by the accumulation of lesions and failure in multiple tissues and organs, including nervous structures (i.e., spinal cord and brain) and digestive organs (i.e., liver, esophagus and colon) [87–90]. As evidenced in our meta-analysis, the cardiac electrocardiographic parameters cannot explain the positive effects of TR inhibitors on mortality reduction.

In general, TR inhibitors attenuated microstructural damage (i.e., necrosis and fibrosis), tissue inflammation, and immunological markers commonly increased in *T. cruzi* infection (i.e., cytokines and specific antibody titers). Taken together, these are important effects potentially related to the reduction of mortality rates in animals treated with TR inhibitors. Attenuation of immunological effectors, especially cytokine and anti-*T. cruzi* antibody levels in animals treated with TR inhibitors (i.e., thioridazine, clomipramine, and tetraamine-based compounds), cannot be interpreted as a disadvantage of chemotherapy that could enhance host susceptibility to infection. On the contrary, reduction in these markers indicated a direct antiparasitic effect of the drugs. Considering parasitological control reflected by low parasitemia levels, attenuation of the immunological response in the face of reduced antigenic load would be expected [39, 83]. This proposition was reinforced by the marked attenuation of heart damage (i.e., myocarditis, necrosis, and fibrosis) in animals treated with TR inhibitors, which indicated that drugs such thioridazine and clomipramine increased host resistance to *T. cruzi* infection. It is broadly recognized that Chagas disease is associated with intense immunological responses that modulates the host-pathogen interaction [91]. At the same time that innate (i.e., macrophages and dendritic and NK cells) and acquired (i.e., T and B lymphocytes) effector agents are essential to reducing parasite survival and replication, exacerbated cellular and humoral responses are detrimental to host cells [24, 92]. Thus, excessive immunological downregulation or upregulation can increase host mortality, in the first case by insufficient defenses to attenuate parasitism and direct organ damage and in the second by inducing tissue lesions in response to massive inflammatory processes [93, 94]. From this perspective, drugs with combined antiparasitic and immunomodulatory properties, including those analyzed in this review, present a great potential for their evaluation in Chagas disease.

Few studies analyzed parasitic load and parasitological cure [43, 69, 95], parameters essential to estimating the efficacy of experimental chemotherapy with greater reliability [69, 96]. As parasites are rarely found in tissues examined by routine histopathological techniques in chronic infections, highly sensitive molecular screenings based on PCR methods are strongly recommended [97–99]. An important disadvantage of PCR measures is the controversial relation between molecular marker levels and real parasitic load [100]. However, this method is currently the gold standard since it was proven that parasite DNA detected by PCR is derived from parasite persistence in host tissues and not from DNA persistence over long periods of time [101]. Although PCR methods are limited to estimating parasitic load, this approach is highly sensitive and reliable as criteria for parasitological cure [99, 102].

Considering the inhibitory potential of TR by all drugs investigated, the molecular affinity of the enzyme for its ligands was evaluated in silico. As the large size of the active site of TR complicates docking studies of the quantitative structure-activity relationship (QSAR), the identification and theoretical development of selective inhibitors from bioinformatics methods is challenging. Our results showed variable molecular affinity between the ligands investigated and TR. In our docking studies, the dibenzazepine clomipramine (1) presented a GlideScore of −3.902 kcal·mol^−1^, which can be correlated with its affinity, although it was lower than that of the substrate trypanothione disulfide (GScore of −8.768 kcal·mol^−1^). Our findings also indicated that the amino acid residue Glu467 of TR is a direct site of interaction with clomipramine by hydrogen bonding. In addition, the phenothiazine thioridazine (2) exhibited a GScore higher than that of clomipramine (−6.099 kcal·mol^−1^) and presented a hydrogen bond interaction with Glu466.

Clomipramine and thioridazine were the most tested TR inhibitors against *T. cruzi* infection *in vivo*. There is evidence that clomipramine [103] and thioridazine [26, 104] inhibit 100% TR activity at low concentrations (Ki = 6.5 *μ*M and 10 *μ*M, respectively). It is worth mentioning that these drugs were primarily developed for the treatment of psychiatric disorders [105]. The dosage and routes of administration of these drugs were based on human equivalent dosages aligned with pharmacological indications for psychiatric disorders [106–108]. Clomipramine and thioridazine do not induce high toxicity [109, 110] and are easily absorbed orally and rapidly distributed through the body [111, 112]. Among their biological properties, they exhibit powerful anti-inflammatory, antitumor, antifungal, antibacterial, anthelmintic, and antiprotozoal activities [39, 113].

The inhibitory activity of tricyclic antidepressants on TR is not completely understood. It has been postulated that these drugs bind to TR with the ring system lodged against the hydrophobic wall formed by Trp21 and Met113 and the aminopropyl side chain extending toward Glu466´ and Glu467´ [114]. More recently, molecular modeling techniques showed that tricyclic drugs, in particular phenothiazine, contained some of the best-fitting probes at the active site of TR and could indeed inhibit the parasite's flavoenzymes [115]. Although there is evidence of parasitological cure after clomipramine and thioridazine administration in *T. cruzi-*infected mice [26, 86], it is still debatable whether this effect is exclusively mediated by TR inhibition and induction of redox imbalance, or other alternative mechanisms. There is evidence that clomipramine and thioridazine also interact with membranes and their components, intracellular proteins, and dopaminergic receptors; inhibit Mg^2+^-dependent ATPase activity; present a strong anticalmodulin activity; induce condensation of cytoplasm organoids; and disrupt mitochondria as well as kinetoplasts [25, 116]. Possibly mediated by the combination of different effects, the lethality induced by clomipramine and thioridazine in trypomastigote and epimastigote forms of *T. cruzi* has been associated with the beneficial effects of chemotherapy in controlling tissue damage and mortality in acute and chronic infections [26, 38, 69].

The psychotropic and other side effects of clomipramine and thioridazine preclude their ample use in the treatment of trypanosome infections [117]. However, employing these drugs as molecular scaffolds to construct more active structures by molecular synthesis could be a reasonable strategy in the development of new antitrypanosomal drugs. As clomipramine and thioridazine have high biodistribution and can cross the blood-brain barrier [26, 110], reservoirs of *T. cruzi* in the central nervous tissues that are not accessible to several antiparasitic drugs could also be treated. This is a notorious pharmacological advantage potentially applicable to overcoming low cure rates, especially in the chronic phases of infection, when *T. cruzi* reservoirs are well established and quiescent [118, 119]. In fact, divergent profiles in the biodistribution of reference drugs (i.e., benznidazole and nifurtimox) have been associated with limited parasite clearance in multiple organs [120, 121]. Moreover, the limited pharmacological efficacy of the reference drugs appears to be associated more significantly with their low bioavailability, a feature that increases the relevance of clomipramine and thioridazine, which exhibit high bioavailability following oral administration [110, 122].

Chemical synthesis was effective in developing two TR inhibitors from phenothiazine's (2-aminodiphenylsulfide [3] and phenothiazine derivative [4]) without significant psychotropic activity [123]. In our molecular docking studies, the GScores of 2-aminodiphenylsulfide (−4.871 kcal·mol^−1^) and the phenothiazine derivative (−5511 kcal·mol^−1^) indicated a small difference in molecular affinity, which was lower than that of trypanothione disulfide. Although the interaction is based on hydrogen bonds, 2-aminodiphenylsulfide and the phenothiazine derivative interact with different amino acid residues in the TR active site, specifically Glu19 and Tyr111, respectively. This interesting finding reinforces the specificity of these molecules in inhibiting TR as molecular interactions occur with amino acid residues that are absent in the human glutathione reductase, the antioxidant enzyme with the highest degree of molecular similarity to TR [124]. Although these molecules induce inhibitory activity against TR (Ki 10 to 25 *μ*M) and *in vitro* cytotoxicity against *T. cruzi* trypomastigotes, *in vivo* toxicity was also identified in a murine model of Chagas disease [123]. However, the low methodological score of this study prompts caution in the interpretation of *in vivo* toxicity results. The main limitations were due to incomplete information on the experimental design of the *in vivo* assays and the absence of key outcome measures (i.e., parasitemia, parasitic load, and markers of toxicity). Therefore, more controlled and comprehensive studies are needed to evaluate the *in vivo* anti-*T. cruzi* potential of phenothiazine derivatives.

An additional strategy in chemical synthesis to potentiate the antiparasitic activity of promising substances is the insertion of metallic components in the primary structure of target molecules [126, 127]. In this sense, two metallodrugs with inhibitory activity against TR were developed and tested *in vivo* by Olmo et al. [43]. These drugs (7 and 8) are manganese coordination complexes containing polyamine ligands, which are also capable of generating highly oxidizing species in *T. cruzi* [43, 128–130]. From our docking model, tetraamine ligand (9) exhibited the best affinity value (GScore −5.400 kcal·mol^−1^), followed by metallodrugs (7) (GScore −4.280 kcal·mol^−1^) and (8) (−3760 kcal·mol^−1^). However, the affinity indexes were not able to explain the antiparasitic activity identified by Olmo et al. [43] *in vitro* or *in vivo*. While metallodrugs (7 and 8) and tetraamine ligand (9) exhibited IC_50_ values of 1.4, 4.4, and 13.6 *μ*M for trypomastigotes *in vitro*, respectively, these drugs reduced parasitemia in 59%, 35%, and 27% in mice infected with *T. cruzi*, respectively. These findings indicate that the antiparasitic effect of these drugs seems to be independent of TR inhibition. This proposition is reinforced by the fact that more than 90% TR inhibition may be required to kill the parasites [104], and these metallodrugs determined only moderate or low TR inhibition *in vitro* (compound 9, 72%, vs. compound 8, 23%) at 100 *μ*M. As identified by Olmo et al. [43], iron superoxide dismutase (Fe-SOD) inhibition and mitochondrial and kinetoplast damage are the potential mechanisms related to *T. cruzi* cytotoxicity. Despite variable antiparasitic effects, both metallodrugs showed promising anti-*T. cruzi* potential, especially considering that they prevented mortality in all treated groups and induced important parasitological cure rates in infected mice (compounds 7, 55%, and 8, 33%) [43].

Beyond synthetic drugs, natural plant products, such as the bisbenzylisoquinoline alkaloids cepharanthine and daphnoline, also showed TR inhibition and were tested in a murine model of *T. cruzi* infection [95]. Docking studies showed higher affinity values for daphnoline (GScore of −6.247 kcal·mol^−1^) than for cepharanthine (GScore of −3.107 kcal·mol^−1^), which was potentially related to two hydrogen bond interactions with the amino acid residues Thr397 and Glu467 in the TR active site. Based on *in vitro* data reported by Fournet et al. [131], TR inhibition and parasite death could not be explained by molecular affinity, since daphnoline and cepharanthine achieved IC_50_ at 50 and 15 *μ*M for TR and 10 and 30 *μ*M for *T. cruzi* trypomastigotes, respectively. In addition, despite excellent TR inhibition, cepharanthine was ineffective at reducing parasitemia or preventing mortality, which occurred in 60% of treated animals, in *T. cruzi*-infected mice. Conversely, daphnoline induced a marked reduction in parasitemia and a high rate of serological cure (60%) compared with the reference drug benznidazole (31.0% cure) [131]. Although a positive relationship between TR inhibition and *in vivo* effects has indicated a promising potential for daphnoline in anti-*T. cruzi* therapy, the trypanocidal mechanisms are possibly not restricted to TR inhibition. Blockage of calcium channels and modulation of the host immune response are also suggested as potential antitrypanosomal mechanisms for this molecule [131], but the evidence is mainly based on *in vitro* models not directly related to *T. cruzi* infection [132, 133]. Further studies are required to evaluate the role of daphnoline in the treatment of Chagas disease.

In this review, we developed an integrated model based on systematic review, meta-analysis, and molecular modeling to gather and analyze the preclinical evidence on the effect of TR inhibitors in *T. cruzi* infections. Our findings indicated that the main methodological limitations of the identified studies were based on recurrent underreporting of the experimental designs and outcome measures, but developing more comprehensive and controlled studies seems to be a feasible task. Our meta-analysis showed a beneficial overall effect in reducing parasitemia and mortality in *T. cruzi*-infected animals. Clomipramine and thioridazine were the most studied drugs, and they did not show protective effects against the occurrence of electrocardiographic abnormalities and affinity and density of cardiac *β*-adrenergic receptors. Although variable enzyme-ligand affinity has been confirmed for all drugs in molecular docking studies, the molecular affinity score was unable to explain TR inhibition and *T. cruzi* death *in vitro* as well as the antiparasitic potential of these drugs when tested in preclinical models of *T. cruzi* infection. The divergence of in silico, *in vitro*, and *in vivo* findings indicated that the anti-*T. cruzi* preclinical effect of the drugs is not be restricted to TR inhibition. Controlled studies are required to determine whether and to what extent additional mechanisms contribute to *T. cruzi* cytotoxicity and trypanocidal effects induced by the TR inhibitors.

## Figures and Tables

**Table 1:** Values of GlideScore (*GScore*), the number of interactions by hydrogen bonds (*Hbond*), van der Waals (*good vdW*), *π*-*π* stacking, and cation *π* between the ligands (1–10), the substrate trypanothione disulfide, and trypanothione reductase.

Ligand	GScore (kcal·mol^−1^)	H bond	Amino acids that perform H bond	Good vdW	*π*-*π* stacking	Cation *π*
Clomipramine (1)	−3.902	1	GLU467	143	0	1
Thioridazine (2)	−6.099	1	GLU466	236	0	0
2-Aminodiphenylsulfide (3)	−4.871	1	GLU19	280	1	0
Phenylthiazine (4)	−5.511	1	TYR111	269	0	0
Cepharanthine (R,S) (5)	−3.107	0	—	257	2	2
Daphnoline (R,S) (6)	−6.247	2	THR397, GLU467	338	0	1
Metallodrug (7)	−4.280	0	—	127	0	0
Metallodrug (8)	−3.760	0	—	236	1	1
Tetraamine ligand (9)	−5.400	3	TYR111, HIS461	280	1	0
Trypanothione disulfide (substrate) (10)	−8.768	9	GLU19, TYR111, THR335, LEU399, GLY459, THR463, GLU466, GLU467	329	0	2

## Supplementary Materials

Supplementary Materials Supplementary Tables S1 to S8: descriptors used in database search and research data extracted from original studies and used in qualitative and quantitative syntheses.

## References

[1] Pereira R. M., Greco G. M. Z., Moreira A. M. (2017). Applicability of plant-based products in the treatment of *Trypanosoma cruzi* and *Trypanosoma brucei* infections: a systematic review of preclinical *in vivo* evidence. *Parasitology*.

[2] WHO (2017). *Chagas Disease*.

[3] Bern C. (2015). Chagas’ disease. *The New England Journal of Medicine*.

[4] Filigheddu M. T., Górgolas M., Ramos J. M. (2017). Orally-transmitted Chagas disease. *Medicina Clínica*.

[5] Murcia L., Carrilero B., Munoz-Davila M. J., Thomas M. C., López M. C., Segovia M. (2013). Risk factors and primary prevention of congenital Chagas disease in a nonendemic country. *Clinical Infectious Diseases*.

[6] Stanaway J. D., Roth G. (2015). The burden of Chagas disease. *Global Heart*.

[7] Novaes R. D., Sartini M. V. P., Rodrigues J. P. F. (2016). Curcumin enhances the anti-*Trypanosoma cruzi* activity of benznidazole-based chemotherapy in acute experimental Chagas disease. *Antimicrobial Agents and Chemotherapy*.

[8] Bocchi E. A. (2013). Heart failure in South America. *Current Cardiology Reviews*.

[9] Freitas H. F. G., Chizzola P. R., Paes Â. T., Lima A. C. P., Mansur A. J. (2005). Risk stratification in a Brazilian hospital-based cohort of 1220 outpatients with heart failure: role of Chagas’ heart disease. *International Journal of Cardiology*.

[10] Andrade J. P. d., Marin Neto J. A., Paola A. A. V. d. (2011). I Diretriz Latino-Americana para o diagnóstico e tratamento da cardiopatia chagásica: resumo executivo. *Arquivos Brasileiros de Cardiologia*.

[11] Nunes M. D. C. P., Barbosa M. M., Ribeiro A. L. P., Fenelon L. M. A., Rocha M. O. C. (2010). Predictors of mortality in patients with dilated cardiomyopathy: relevance of chagas disease as an etiological factor. *Revista Española de Cardiología*.

[12] Lee B. Y., Bacon K. M., Bottazzi M. E., Hotez P. J. (2013). Global economic burden of Chagas disease: a computational simulation model. *Lancet Infectious Diseases*.

[13] Urbina J. A., Docampo R. (2003). Specific chemotherapy of Chagas disease: controversies and advances. *Trends in Parasitology*.

[14] Urbina J. A. (2010). Specific chemotherapy of Chagas disease: relevance, current limitations and new approaches. *Acta Tropica*.

[15] Santos E. C., Novaes R. D., Cupertino M. C. (2015). Concomitant benznidazole and suramin chemotherapy in mice infected with a virulent strain of *Trypanosoma cruzi*. *Antimicrobial Agents and Chemotherapy*.

[16] Khan M. O. F. (2007). Trypanothione reductase: a viable chemotherapeutic target for antitrypanosomal and antileishmanial drug design. *Drug Target Insights*.

[17] Chirac P., Torreele E. (2006). Global framework on essential health R&D. *The Lancet*.

[18] Goupil L. S., McKerrow J. H. (2014). Introduction: drug discovery and development for neglected diseases. *Chemical Reviews*.

[19] Ferreira L. G., Andricopulo A. D. (2016). Drug repositioning approaches to parasitic diseases: a medicinal chemistry perspective. *Drug Discovery Today*.

[20] Turrens J. F. (2004). Oxidative stress and antioxidant defenses: a target for the treatment of diseases caused by parasitic protozoa. *Molecular Aspects of Medicine*.

[21] Krauth-Siegel R. L., Comini M. A. (2008). Redox control in trypanosomatids, parasitic protozoa with trypanothione-based thiol metabolism. *Biochimica et Biophysica Acta-General Subjects*.

[22] Hunter W. N., Bailey S., Habash J. (1992). Active site of trypanothione reductase. *Journal of Molecular Biology*.

[23] Sorci G., Faivre B. (2009). Inflammation and oxidative stress in vertebrate host-parasite systems. *Philosophical Transactions of the Royal Society B: Biological Sciences*.

[24] Machado F. S., Dutra W. O., Esper L. (2012). Current understanding of immunity to *Trypanosoma cruzi* infection and pathogenesis of Chagas disease. *Seminars in Immunopathology*.

[25] Rivarola H. W., Paglini-Oliva P. A. (2002). *Trypanosoma cruzi* trypanothione reductase inhibitors: Phenothiazines and related compounds modify experimental Chagas’ disease evolution. *Current Drug Targets-Cardiovascular & Hematological Disorders*.

[26] Lo Presti M. S., Bazán P. C., Strauss M., Báez A. L., Rivarola H. W., Paglini-Oliva P. A. (2015). Trypanothione reductase inhibitors: overview of the action of thioridazine in different stages of Chagas disease. *Acta Tropica*.

[27] Martyn D. C., Jones D. C., Fairlamb A. H., Clardy J. (2007). High-throughput screening affords novel and selective trypanothione reductase inhibitors with anti-trypanosomal activity. *Bioorganic & Medicinal Chemistry Letters*.

[28] Beltran-Hortelano I., Perez-Silanes S., Galiano S. (2017). Trypanothione reductase and superoxide dismutase as current drug targets for *Trypanosoma cruzi*: an overview of compounds with activity against Chagas disease. *Current Medicinal Chemistry*.

[29] Chacón-Vargas K., Nogueda-Torres B., Sánchez-Torres L. (2017). Trypanocidal activity of quinoxaline 1,4 Di-N-oxide derivatives as trypanothione reductase inhibitors. *Molecules*.

[30] Moher D., Liberati A., Tetzlaff J., Altman D. G., The PRISMA Group (2009). Preferred reporting items for systematic reviews and meta-analyses: the PRISMA statement. *PLoS Medicine*.

[31] Hooijmans C. R., Tillema A., Leenaars M., Ritskes-Hoitinga M. (2010). Enhancing search efficiency by means of a search filter for finding all studies on animal experimentation in PubMed. *Laboratory Animals*.

[32] Bond C. S., Zhang Y., Berriman M., Cunningham M. L., Fairlamb A. H., Hunter W. N. (1999). Crystal structure of *Trypanosoma cruzi* trypanothione reductase in complex with trypanothione, and the structure-based discovery of new natural product inhibitors. *Structure*.

[33] Kilkenny C., Browne W., Cuthill I. C., Emerson M., Altman D. G., NC3Rs Reporting Guidelines Working Group (2010). Animal research: reporting in vivo experiments: the ARRIVE guidelines. *British Journal of Pharmacology*.

[34] DerSimonian R., Laird N. (1986). Meta-analysis in clinical trials. *Controlled Clinical Trials*.

[35] Higgins J. P. T., Thompson S. G., Deeks J. J., Altman D. G. (2003). Measuring inconsistency in meta-analyses. *British Medical Journal*.

[36] Hooijmans C. R., Pasker-De Jong P. C. M., De Vries R. B. M., Ritskes-Hoitinga M. (2012). The effects of long-term omega-3 fatty acid supplementation on cognition and Alzheimer’s pathology in animal models of Alzheimer’s disease: a systematic review and meta-analysis. *Journal of Alzheimer's Disease*.

[37] Lo Presti M. S., Rivarola H. W., Bustamante J. M. (2004). Thioridazine treatment prevents cardiopathy in *Trypanosoma cruzi* infected mice. *International Journal of Antimicrobial Agents*.

[38] Rivarola H. W., Bustamante J. M., Lo Presti S. (2005). *Trypanosoma cruzi*: chemotherapeutic effects of clomipramine in mice infected with an isolate obtained from an endemic area. *Experimental Parasitology*.

[39] Fauro R., Presti S. L., Bazan C. (2013). Use of clomipramine as chemotherapy of the chronic phase of Chagas disease. *Parasitology*.

[40] Strauss M., Lo Presti M. S., Bazán P. C. (2013). Clomipramine and benznidazole association for the treatment of acute experimental *Trypanosoma cruzi* infection. *Parasitology International*.

[41] Rivarola H. W., Fernández A. R., Enders J. E. (1999). Thioridazine treatment modifies the evolution of *Trypanosoma cruzi* infection in mice. *Annals of Tropical Medicine & Parasitology*.

[42] Gobbi P., Baez A., Lo Presti M. S. (2010). Association of clomipramine and allopurinol for the treatment of the experimental infection with *Trypanosoma cruzi*. *Parasitology Research*.

[43] Olmo F., Cussó O., Marín C. (2016). In vitro and in vivo identification of tetradentated polyamine complexes as highly efficient metallodrugs against *Trypanosoma cruzi*. *Experimental Parasitology*.

[44] Novaes R. D., Gonçalves R. V., Penitente A. R. (2016). Modulation of inflammatory and oxidative status by exercise attenuates cardiac morphofunctional remodeling in experimental Chagas cardiomyopathy. *Life Sciences*.

[45] Novaes R. D., Santos E. C., Fialho M. D. C. Q. (2017). Nonsteroidal anti-inflammatory is more effective than anti-oxidant therapy in counteracting oxidative/nitrosative stress and heart disease in T. cruzi-infected mice. *Parasitology*.

[46] Novaes R. D., Gonçalves R. V., Penitente A. R. (2017). Parasite control and skeletal myositis in *Trypanosoma cruzi*-infected and exercised rats. *Acta Tropica*.

[47] Novaes R. D., Santos E. C., Cupertino M. C. (2015). *Trypanosoma cruzi* infection and benznidazole therapy independently stimulate oxidative status and structural pathological remodeling of the liver tissue in mice. *Parasitology Research*.

[48] Triquell M. F., Díaz-Luján C., Romanini M. C. (2018). Nitric oxide synthase and oxidative-nitrosative stress play a key role in placental infection by *Trypanosoma cruzi*. *American Journal of Reproductive Immunology*.

[49] Gupta S., Wen J.-J., Garg N. J. (2009). Oxidative stress in Chagas disease. *Interdisciplinary Perspectives on Infectious Diseases*.

[50] Rodrigues J. P. F., Caldas I. S., Gonçalves R. V., Almeida L. A., Souza R. L. M., Novaes R. D. (2017). S. mansoni-T. cruzi co-infection modulates arginase-1/iNOS expression, liver and heart disease in mice. *Nitric Oxide*.

[51] Gupta S., Bhatia V., Wen J.-J., Wu Y., Huang M. H., Garg N. J. (2009). *Trypanosoma cruzi* infection disturbs mitochondrial membrane potential and ROS production rate in cardiomyocytes. *Free Radical Biology & Medicine*.

[52] Zoltowski A. P. C., Costa A. B., Teixeira M. A. P., Koller S. H. (2014). Methodological quality of systematic reviews in Brazilian psychology journals quality of systematic reviews in psychology. *Psicologia: Teoria e Pesquisa*.

[53] da Silva Filho C. R., Saconato H., Conterno L. O., Marques I., Atallah Á. N. (2005). Assessment of clinical trial quality and its impact on meta-analyses. *Revista de Saúde Pública*.

[54] Curtis M. J., Bond R. A., Spina D. (2015). Experimental design and analysis and their reporting: new guidance for publication in BJP. *British Journal of Pharmacology*.

[55] McGrath J. C., Lilley E. (2015). Implementing guidelines on reporting research using animals (ARRIVE etc.): new requirements for publication in BJP. *British Journal of Pharmacology*.

[56] Andrade L. O., Machado C. R. S., Chiari E., Pena S. D. J., Macedo A. M. (2002). *Trypanosoma cruzi*: role of host genetic background in the differential tissue distribution of parasite clonal populations. *Experimental Parasitology*.

[57] León C. M., Montilla M., Vanegas R., Castillo M., Parra E., Ramírez J. D. (2017). Murine models susceptibility to distinct *Trypanosoma cruzi* I genotypes infection. *Parasitology*.

[58] Jelicks L. A., Tanowitz H. B. (2011). Advances in imaging of animal models of Chagas disease. *Advances in Parasitology*.

[59] Manoel-Caetano F. D. S., Silva A. E. (2007). Implications of genetic variability of *Trypanosoma cruzi* for the pathogenesis of Chagas disease. *Cadernos de Saúde Pública*.

[60] Chatelain E., Konar N. (2015). Translational challenges of animal models in Chagas disease drug development: a review. *Drug Design, Development and Therapy*.

[61] Silva G. K., Cunha L. D., Horta C. V. (2013). A parent-of-origin effect determines the susceptibility of a non-informative F1 population to *Trypanosoma cruzi* infection in vivo. *PLoS One*.

[62] Piacenza L., Peluffo G., Alvarez M. N., Martínez A., Radi R. (2013). *Trypanosoma cruzi* antioxidant enzymes as virulence factors in Chagas disease. *Antioxidants & Redox Signaling*.

[63] da Silva Guerreiro M. L., Morais I. R. B., Andrade S. G. (2015). Immunological response to re-infections with clones of the Colombian strain of *Trypanosoma cruzi* with different degrees of virulence: influence on pathological features during chronic infection in mice. *Memórias do Instituto Oswaldo Cruz*.

[64] Dohoo I., Stryhn H., Sanchez J. (2007). Evaluation of underlying risk as a source of heterogeneity in meta-analyses: a simulation study of Bayesian and frequentist implementations of three models. *Preventive Veterinary Medicine*.

[65] Lin L., Chu H., Hodges J. S. (2017). Alternative measures of between-study heterogeneity in meta-analysis: reducing the impact of outlying studies. *Biometrics*.

[66] Bazán P. C., Lo Presti M. S., Rivarola H. W. (2008). Chemotherapy of chronic indeterminate Chagas disease: a novel approach to treatment. *Parasitology Research*.

[67] Miyazaki Y., Hamano S., Wang S., Shimanoe Y., Iwakura Y., Yoshida H. (2010). IL-17 is necessary for host protection against acute-phase *Trypanosoma cruzi* infection. *Journal of Immunology*.

[68] Sanches T. L. M., Cunha L. D., Silva G. K., Guedes P. M. M., Silva J. S., Zamboni D. S. (2014). The use of a heterogeneously controlled mouse population reveals a significant correlation of acute phase parasitemia with mortality in Chagas disease. *PLoS One*.

[69] Bazán P. C., Lo Presti M. S., Strauss M. (2016). Quantitative PCR and unconventional serological methods to evaluate clomipramine treatment effectiveness in experimental *Trypanosoma cruzi* infection. *Experimental and Molecular Pathology*.

[70] Vázquez K., Paulino M., Salas C. O., Zarate-Ramos J. J., Vera B., Rivera G. (2017). Trypanothione reductase: a target for the development of anti-*Trypanosoma cruzi* drugs. *Mini-Reviews in Medicinal Chemistry*.

[71] Alviano D. S., Barreto A. L. S., Dias F. . A. (2012). Conventional therapy and promising plant-derived compounds against trypanosomatid parasites. *Frontiers in Microbiology*.

[72] Kelly M. J., Wilkinson S. R. (2013). Mechanisms of resistance to antiparasitic drugs in *Trypanosoma cruzi*. Correlations between genotype and resistance. *Revista Española de Salud Publica*.

[73] Santos F. L. N., Celedon P. A. F., Zanchin N. I. T. (2016). Performance assessment of four chimeric *Trypanosoma cruzi* antigens based on antigen-antibody detection for diagnosis of chronic Chagas disease. *PLoS One*.

[74] Rojo G., Sandoval-Rodríguez A., López A. (2017). Within-host temporal fluctuations of *Trypanosoma cruzi* discrete typing units: the case of the wild reservoir rodent Octodon degus. *Parasites & Vectors*.

[75] Romano M. M. D., Moreira H. T., Schmidt A., Maciel B. C., Marin-Neto J. A. (2017). Imaging diagnosis of right ventricle involvement in Chagas cardiomyopathy. *BioMed Research International*.

[76] Traina M. I., Hernandez S., Sanchez D. R. (2017). Prevalence of Chagas disease in a U.S. population of Latin American immigrants with conduction abnormalities on electrocardiogram. *PLoS Neglected Tropical Diseases*.

[77] Rassi A., Rassi A., Marin-Neto J. A. (2010). Chagas disease. *The Lancet*.

[78] Rossi M. A., Tanowitz H. B., Malvestio L. M. (2010). Coronary microvascular disease in chronic Chagas cardiomyopathy including an overview on history, pathology, and other proposed pathogenic mechanisms. *PLoS Neglected Tropical Diseases*.

[79] Prado C. M., Celes M. R. N., Malvestio L. M. (2012). Early dystrophin disruption in the pathogenesis of experimental chronic Chagas cardiomyopathy. *Microbes and Infection*.

[80] Paglini-Oliva P., Lo Presti S. M., Rivarola H. W., Shah M. M. (2012). Electrocardiography as a diagnostic method for chagas disease in patients and experimental models. *Parasitology*.

[81] Jahns R., Boivin V., Lohse M. (2006). *β*1-Adrenergic receptor function, autoimmunity, and pathogenesis of dilated cardiomyopathy. *Trends in Cardiovascular Medicine*.

[82] Labovsky V., Smulski C. R., Gómez K., Levy G., Levin M. J. (2007). Anti-*β*1-adrenergic receptor autoantibodies in patients with chronic Chagas heart disease. *Clinical and Experimental Immunology*.

[83] Rivarola H. W., Fernandez A. R., Enders J. E., Fretes R., Gea S., Paglini-Oliva P. (2001). Effects of clomipramine on *Trypanosoma cruzi* infection in mice. *Transactions of the Royal Society of Tropical Medicine and Hygiene*.

[84] Sterin-Borda L., Borda E. (2000). Role of neurotransmitter autoantibodies in the pathogenesis of Chagasic peripheral dysautonomia. *Annals of the New York Academy of Sciences*.

[85] Nussinovitch U., Shoenfeld Y. (2013). The clinical significance of anti-beta-1 adrenergic receptor autoantibodies in cardiac disease. *Clinical Reviews in Allergy & Immunology*.

[86] García M. C., Ponce N. E., Sanmarco L. M., Manzo R. H., Jimenez-Kairuz A. F., Aoki M. P. (2016). Clomipramine and benznidazole act synergistically and ameliorate the outcome of experimental Chagas disease. *Antimicrobial Agents and Chemotherapy*.

[87] Higuchi M., Benvenuti L. A., Martins Reis M., Metzger M. (2003). Pathophysiology of the heart in Chagas’ disease: current status and new developments. *Cardiovascular Research*.

[88] Marin-Neto J. A., Cunha-Neto E., Maciel B. C., Simoes M. V. (2007). Pathogenesis of chronic Chagas heart disease. *Circulation*.

[89] Biolo A., Ribeiro A. L., Clausell N. (2010). Chagas cardiomyopathy--where do we stand after a hundred years?. *Progress in Cardiovascular Diseases*.

[90] Campos C. F., Cangussú S. D., Duz A. L. C. (2016). Enteric neuronal damage, intramuscular denervation and smooth muscle phenotype changes as mechanisms of Chagasic megacolon: evidence from a long-term murine model of Tripanosoma cruzi infection. *PLoS One*.

[91] Cuervo H., Guerrero N. A., Carbajosa S. (2011). Myeloid-derived suppressor cells infiltrate the heart in acute *Trypanosoma cruzi* infection. *Journal of Immunology*.

[92] Cardoso M. S., Reis-Cunha J. L., Bartholomeu D. C. (2016). Evasion of the immune response by *Trypanosoma cruzi* during acute infection. *Frontiers in Immunology*.

[93] McAuley J. L., Kedzierska K., Brown L. E., Shanks G. D. (2015). Host immunological factors enhancing mortality of young adults during the 1918 influenza pandemic. *Frontiers in Immunology*.

[94] Cardoso R., Garcia D., Fernandes G. (2016). The prevalence of atrial fibrillation and conduction abnormalities in Chagas’ disease: a meta-analysis. *Journal of Cardiovascular Electrophysiology*.

[95] Fournet A., Rojas de Arias A., Ferreira M. E. (2000). Efficacy of the bisbenzylisoquinoline alkaloids in acute and chronic *Trypanosoma cruzi* murine model. *International Journal of Antimicrobial Agents*.

[96] Guedes P. M., Silva G. K., Gutierrez F. R., Silva J. S. (2011). Current status of Chagas disease chemotherapy. *Expert Review of Anti-infective Therapy*.

[97] Vago A. R., Andrade L. O., Leite A. A. (2000). Genetic characterization of *Trypanosoma cruzi* directly from tissues of patients with chronic Chagas disease. *The American Journal of Pathology*.

[98] Schijman A. G., Bisio M., Orellana L. (2011). International study to evaluate PCR methods for detection of *Trypanosoma cruzi* DNA in blood samples from Chagas disease patients. *PLoS Neglected Tropical Diseases*.

[99] Caldas S., Caldas I. S., Diniz L. . F. (2012). Real-time PCR strategy for parasite quantification in blood and tissue samples of experimental *Trypanosoma cruzi* infection. *Acta Tropica*.

[100] Piron M., Fisa R., Casamitjana N. (2007). Development of a real-time PCR assay for *Trypanosoma cruzi* detection in blood samples. *Acta Tropica*.

[101] Marcon G. E. B., Albuquerque D. M. ., Batista A. M. (2011). *Trypanosoma cruzi*: parasite persistence in tissues in chronic Chagasic Brazilian patients. *Memórias do Instituto Oswaldo Cruz*.

[102] Duffy T., Bisio M., Altcheh J. (2009). Accurate real-time PCR strategy for monitoring bloodstream parasitic loads in Chagas disease patients. *PLoS Neglected Tropical Diseases*.

[103] Benson T. J., McKie J. H., Garforth J., Borges A., Fairlamb A. H., Douglas K. T. (1992). Rationally designed selective inhibitors of trypanothione reductase. Phenothiazines and related tricyclics as lead structures. *Biochemical Journal*.

[104] Fairlamb A. H. (1999). Future prospects for the chemotherapy of Chagas’ disease. *Medicina*.

[105] Gutierrez-Correa J., Fairlamb A. H., Stoppani A. O. M. (2001). *Trypanosoma cruzi* trypanothione reductase is inactivated by peroxidase-generated phenothiazine cationic radicals. *Free Radical Research*.

[106] Contrera J. F., Matthews E. J., Kruhlak N. L., Benz R. D. (2004). Estimating the safe starting dose in phase I clinical trials and no observed effect level based on QSAR modeling of the human maximum recommended daily dose. *Regulatory Toxicology and Pharmacology*.

[107] Shin J., Seol I., Son C. (2010). Interpretation of animal dose and human equivalent dose for drug development. *The Journal of Korean Oriental Medicine*.

[108] Nair A. B., Jacob S. (2016). A simple practice guide for dose conversion between animals and human. *Journal of Basic and Clinical Pharmacy*.

[109] Balant-Gorgia A. E., Gex-Fabry M., Balant L. P. (1991). Clinical pharmacokinetics of clomipramine. *Clinical Pharmacokinetics*.

[110] Thanacoody H. K. R. (2007). Thioridazine: resurrection as an antimicrobial agent?. *British Journal of Clinical Pharmacology*.

[111] Daniel W. A., Syrek M., Haduch A., Wójcikowski J. (2000). Pharmacokinetics and metabolism of thioridazine during co-administration of tricyclic antidepressants. *British Journal of Clinical Pharmacology*.

[112] Kobuchi S., Fukushima K., Shibata M., Ito Y., Sugioka N., Takada K. (2011). Pharmacokinetics of clomipramine, an antidepressant, in poloxamer 407-induced hyperlipidaemic model rats. *Journal of Pharmacy and Pharmacology*.

[113] Amaral L., Viveiros M., Kristiansen J. E. (2001). Phenothiazines: potential alternatives for the management of antibiotic resistant infections of tuberculosis and malaria in developing countries. *Tropical Medicine and International Health*.

[114] Krauth-Siegel R. L., Inhoff O. (2003). Parasite-specific trypanothione reductase as a drug target molecule. *Parasitology Research*.

[115] Paulino M., Iribarne F., Dubin M., Aguilera-Morales S., Tapia O., Stoppani A. (2005). The chemotherapy of Chagas disease: an overview. *Mini-Reviews in Medicinal Chemistry*.

[116] Paglini-Oliva P., Rivarola H. (2003). Central nervous system agents used as *Trypanosoma cruzi* infection chemotherapy: phenothiazines and related compounds. *Current Medicinal Chemistry -Anti-Infective Agents*.

[117] O’Sullivan M. C., Durham T. B., Valdes H. E. (2015). Dibenzosuberyl substituted polyamines and analogs of clomipramine as effective inhibitors of trypanothione reductase: molecular docking, and assessment of trypanocidal activities. *Bioorganic & Medicinal Chemistry*.

[118] Pittella J. E. H. (2009). Central nervous system involvement in Chagas disease: a hundred-year-old history. *Transactions of the Royal Society of Tropical Medicine and Hygiene*.

[119] da Silva A. A., Pereira G. V., de Souza A. S., Silva R. R., Rocha M. S., Lannes-Vieira J. (2010). *Trypanosoma cruzi*-induced central nervous system alterations: from the entry of inflammatory cells to potential cognitive and psychiatric abnormalities. *Journal of Neuroparasitology*.

[120] Esperandim V. R., da Silva Ferreira D., Saraiva J. (2010). Reduction of parasitism tissue by treatment of mice chronically infected with *Trypanosoma cruzi* with lignano lactones. *Parasitology Research*.

[121] Bahia M. T., Diniz L. D. F., Mosqueira V. C. F. (2014). Therapeutical approaches under investigation for treatment of Chagas disease. *Expert Opinion on Investigational Drugs*.

[122] Lainesse C., Frank D., Meucci V., Intorre L., Soldani G., Doucet M. (2006). Pharmacokinetics of clomipramine and desmethylclomipramine after single-dose intravenous and oral administrations in cats. *Journal of Veterinary Pharmacology and Therapeutics*.

[123] Fernandez-Gomez R., Moutiez M., Aumercier M. (1995). 2-Amino diphenylsulfides as new inhibitors of trypanothione reductase. *International Journal of Antimicrobial Agents*.

[124] Iribarne F., Paulino M., Aguilera S., Tapia O. (2009). Assaying phenothiazine derivatives as trypanothione reductase and glutathione reductase inhibitors by theoretical docking and molecular dynamics studies. *Journal of Molecular Graphics and Modelling*.

[125] Baillet S., Buisine E., Horvath D., Maes L., Bonnet B., Sergheraert C. (1996). 2-Amino diphenylsulfides as inhibitors of trypanothione reductase: modification of the side chain. *Bioorganic & Medicinal Chemistry*.

[126] Navarro M., Gabbiani C., Messori L., Gambino D. (2010). Metal-based drugs for malaria, trypanosomiasis and leishmaniasis: recent achievements and perspectives. *Drug Discovery Today*.

[127] Barry N. P. E., Sadler P. J. (2013). Exploration of the medical periodic table: towards new targets. *Chemical Communications*.

[128] Company A., Prat I., Frisch J. R. (2011). Modeling the cis-oxo-labile binding site motif of non-heme iron oxygenases: water exchange and oxidation reactivity of a non-heme iron (IV)-oxo compound bearing a tripodal tetradentate ligand. *Chemistry*.

[129] Garcia-Bosch I., Gómez L., Polo A., Ribas X., Costas M. (2012). Stereoselective epoxidation of alkenes with hydrogen peroxide using a bipyrrolidine-based family of manganese complexes. *Advanced Synthesis & Catalysis*.

[130] Cussó O., Garcia-Bosch I., Ribas X., Lloret-Fillol J., Costas M. (2013). Asymmetric epoxidation with H_2_O_2_ by manipulating the electronic properties of non-heme iron catalysts. *Journal of the American Chemical Society*.

[131] Fournet A., Inchausti A., Yaluff G. (1998). Trypanocidal bisbenzylisoquinoline alkaloids are inhibitors of trypanothione reductase. *Journal of Enzyme Inhibition*.

[132] Kondo Y., Takano F., Hojo H. (1993). Inhibitory effect of bisbenzylisoquinoline alkaloids on nitric oxide production in activated macrophages. *Biochemical Pharmacology*.

[133] Seow W. K., Nakamura K., Sugimura Y. (1993). Inhibitory effects of bisbenzylisoquinolines on synthesis of the inflammatory cytokines interleukin-1 and tumour necrosis factor-alpha. *Mediators of Inflammation*.

