# Supplementary material for: Relevance of Trypanothione Reductase Inhibitors on Trypanosoma cruzi Infection: A Systematic Review, Meta-Analysis, and In Silico Integrated Approach
Source: Oxid Med Cell Longev. 2018 Oct 24;2018:8676578. doi: 10.1155/2018/8676578 (PMC6220389; doi:10.1155/2018/8676578)
Supplement: Supplementary Materials — Supplementary Tables S1 to S8: descriptors used in database search and research data extracted from original studies and used in qualitative and quantitative syntheses. [file 8676578.f1.pdf]

Table S1. Complete search strategy with search filters and number of studies recovered in databases PubMed-Medline, Scopus and Web of Sciences.

PubMed-MEDLINE – Search filters	***Retrieved records***
**#1 Disease:** (“Chagas disease”[TIAB] OR “American trypanosomiasis”[TIAB] OR “Trypanosoma cruzi”[TIAB] OR “Trypanosoma cruzus”[TIAB])	17.002
**#2 Therapeutic target / intervention:** (“trypanothione reductase”[TIAB] OR “trypanothione reductase inhibitors”[TIAB])	378
**#3 First animal filter:** ("animal experimentation"[MeSH Terms] OR "models, animal"[MeSH Terms] OR "invertebrates"[MeSH Terms] OR "Animals"[Mesh:noexp] OR "animal population groups"[MeSH Terms] OR "chordata"[MeSH Terms:noexp] OR "chordata, nonvertebrate"[MeSH Terms] OR "vertebrates"[MeSH Terms:noexp] OR "amphibians"[MeSH Terms] OR "birds"[MeSH Terms] OR "fishes"[MeSH Terms] OR "reptiles"[MeSH Terms] OR "mammals"[MeSH Terms:noexp] OR "primates"[MeSH Terms:noexp] OR "artiodactyla"[MeSH Terms] OR "carnivora"[MeSH Terms] OR "cetacea"[MeSH Terms] OR "chiroptera"[MeSH Terms] OR "elephants"[MeSH Terms] OR "hyraxes"[MeSH Terms] OR "insectivora"[MeSH Terms] OR "lagomorpha"[MeSH Terms] OR "marsupialia"[MeSH Terms] OR "monotremata"[MeSH Terms] OR "perissodactyla"[MeSH Terms] OR "rodentia"[MeSH Terms] OR "scandentia"[MeSH Terms] OR "sirenia"[MeSH Terms] OR "xenarthra"[MeSH Terms] OR "haplorhini"[MeSH Terms:noexp] OR "strepsirhini"[MeSH Terms] OR "platyrrhini"[MeSH Terms] OR "tarsii"[MeSH Terms] OR "catarrhini"[MeSH Terms:noexp] OR "cercopithecidae"[MeSH Terms] OR "hylobatidae"[MeSH Terms] OR "hominidae"[MeSH Terms:noexp] OR "gorilla gorilla"[MeSH Terms] OR "pan paniscus"[MeSH Terms] OR "pan troglodytes"[MeSH Terms] OR "pongo pygmaeus"[MeSH Terms])	5.990.987

#4 Second animal filter:

((animals[tiab] OR animal[tiab] OR mice[Tiab] OR mus[Tiab] OR mouse[Tiab] OR murine[Tiab] OR woodmouse[tiab] OR rats[Tiab] OR rat[Tiab] OR murinae[Tiab] OR muridae[Tiab] OR cottonrat[tiab] OR cottonrats[tiab] OR hamster[tiab] OR hamsters[tiab] OR cricetinae[tiab] OR rodentia[Tiab] OR rodent[Tiab] OR rodents[Tiab] OR pigs[Tiab] OR pig[Tiab] OR swine[tiab] OR swines[tiab] OR piglets[tiab] OR piglet[tiab] OR boar[tiab] OR boars[tiab] OR "sus scrofa"[tiab] OR ferrets[tiab] OR ferret[tiab] OR polecat[tiab] OR polecats[tiab] OR "mustela putorius"[tiab] OR "guinea pigs"[Tiab] OR "guinea pig"[Tiab] OR cavia[Tiab] OR callithrix[Tiab] OR marmoset[Tiab] OR marmosets[Tiab] OR cebuella[Tiab] OR hapale[Tiab] OR octodon[Tiab] OR chinchilla[Tiab] OR chinchillas[Tiab] OR gerbillinae[Tiab] OR gerbil[Tiab] OR gerbils[Tiab] OR jird[Tiab] OR jirds[Tiab] OR merione[Tiab] OR meriones[Tiab] OR rabbits[Tiab] OR rabbit[Tiab] OR hares[Tiab] OR hare[Tiab] OR diptera[Tiab] OR flies[Tiab] OR fly[Tiab] OR dipteral[Tiab] OR drosphila[Tiab] OR drosophilidae[Tiab] OR cats[Tiab] OR cat[Tiab] OR carus[Tiab] OR felis[Tiab] OR nematoda[Tiab] OR nematode[Tiab] OR nematoda[Tiab] OR nematode[Tiab] OR nematodes[Tiab] OR sipunculida[Tiab] OR dogs[Tiab] OR dog[Tiab] OR canine[Tiab] OR canines[Tiab] OR canis[Tiab] OR sheep[Tiab] OR sheeps[Tiab] OR mouflon[Tiab] OR mouflons[Tiab] OR ovis[Tiab] OR goats[Tiab] OR goat[Tiab] OR capra[Tiab] OR capras[Tiab] OR rupicapra[Tiab] OR chamois[Tiab] OR haplorhini[Tiab] OR monkey[Tiab] OR monkeys[Tiab] OR anthropoidea[Tiab] OR anthropoids[Tiab] OR saguinus[Tiab] OR tamarin[Tiab] OR tamarins[Tiab] OR leontopithecus[Tiab] OR hominidae[Tiab] OR ape[Tiab] OR apes[Tiab] OR pan[Tiab] OR paniscus[Tiab] OR "pan paniscus"[Tiab] OR bonobo[Tiab] OR bonobos[Tiab] OR troglodytes[Tiab] OR "pan troglodytes"[Tiab] OR gibbon[Tiab] OR gibbons[Tiab] OR siamang[Tiab] OR siamangs[Tiab] OR nomascus[Tiab] OR symphalangus[Tiab] OR chimpanzee[Tiab] OR chimpanzees[Tiab] OR prosimians[Tiab] OR "bush baby"[Tiab] OR prosimian[Tiab] OR bush babies[Tiab] OR galagos[Tiab] OR galago[Tiab]

288.098

OR pongidae[Tiab] OR gorilla[Tiab] OR gorillas[Tiab] OR pongo[Tiab] OR pygmaeus[Tiab] OR "pongo pygmaeus"[Tiab] OR orangutans[Tiab] OR pygmaeus[Tiab] OR lemur[Tiab] OR lemurs[Tiab] OR lemuridae[Tiab] OR horse[Tiab] OR horses[Tiab] OR pongo[Tiab] OR equus[Tiab] OR cow[Tiab] OR calf[Tiab] OR bull[Tiab] OR chicken[Tiab] OR chickens[Tiab] OR gallus[Tiab] OR quail[Tiab] OR bird[Tiab] OR birds[Tiab] OR quails[Tiab] OR poultry[Tiab] OR poultries[Tiab] OR fowl[Tiab] OR fowls[Tiab] OR reptile[Tiab] OR reptilia[Tiab] OR reptiles[Tiab] OR snakes[Tiab] OR snake[Tiab] OR lizard[Tiab] OR lizards[Tiab] OR alligator[Tiab] OR alligators[Tiab] OR crocodile[Tiab] OR crocodiles[Tiab] OR turtle[Tiab] OR turtles[Tiab] OR amphibian[Tiab] OR amphibians[Tiab] OR amphibia[Tiab] OR frog[Tiab] OR frogs[Tiab] OR bombina[Tiab] OR salientia[Tiab] OR toad[Tiab] OR toads[Tiab] OR "epidalea calamita"[Tiab] OR salamander[Tiab] OR salamanders[Tiab] OR eel[Tiab] OR eels[Tiab] OR fish[Tiab] OR fishes[Tiab] OR pisces[Tiab] OR catfish[Tiab] OR catfishes[Tiab] OR siluriformes[Tiab] OR arius[Tiab] OR heteropneustes[Tiab] OR sheatfish[Tiab] OR perch[Tiab] OR perches[Tiab] OR percidae[Tiab] OR perca[Tiab] OR trout[Tiab] OR trouts[Tiab] OR char[Tiab] OR chars[Tiab] OR salvelinus[Tiab] OR "fathead minnow"[Tiab] OR minnow[Tiab] OR cyprinidae[Tiab] OR carps[Tiab] OR carp[Tiab] OR zebrafish[Tiab] OR zebrafishes[Tiab] OR goldfish[Tiab] OR goldfishes[Tiab] OR guppy[Tiab] OR guppies[Tiab] OR chub[Tiab] OR chubs[Tiab] OR tinca[Tiab] OR barbels[Tiab] OR barbus[Tiab] OR pimephales[Tiab] OR promelas[Tiab] OR "poecilia reticulata"[Tiab] OR mullet[Tiab] OR mullets[Tiab] OR seahorse[Tiab] OR seahorses[Tiab] OR mugil curema[Tiab] OR atlantic cod[Tiab] OR shark[Tiab] OR sharks[Tiab] OR catshark[Tiab] OR anguilla[Tiab] OR salmonid[Tiab] OR salmonids[Tiab] OR whitefish[Tiab] OR whitefishes[Tiab] OR salmon[Tiab] OR salmons[Tiab] OR sole[Tiab] OR solea[Tiab] OR "sea lamprey"[Tiab] OR lamprey[Tiab] OR lampreys[Tiab] OR pumpkinseed[Tiab] OR sunfish[Tiab] OR sunfishes[Tiab] OR tilapia[Tiab] OR tilapias[Tiab] OR turbot[Tiab] OR turbots[Tiab] OR flatfish[Tiab] OR flatfishes[Tiab] OR sciuridae[Tiab] OR squirrel[Tiab] OR squirrels[Tiab]

OR chipmunk[Tiab] OR chipmunks[Tiab] OR suslik[Tiab] OR susliks[Tiab] OR vole[Tiab] OR voles[Tiab] OR lemming[Tiab] OR lemmings[Tiab] OR muskrat[Tiab] OR muskrats[Tiab] OR lemmus[Tiab] OR otter[Tiab] OR otters[Tiab] OR marten[Tiab] OR martens[Tiab] OR martes[Tiab] OR weasel[Tiab] OR badger[Tiab] OR badgers[Tiab] OR ermine[Tiab] OR mink[Tiab] OR minks[Tiab] OR sable[Tiab] OR sables[Tiab] OR gulo[Tiab] OR gulos[Tiab] OR wolverine[Tiab] OR wolverines[Tiab] OR minks[Tiab] OR mustela[Tiab] OR llama[Tiab] OR llamas[Tiab] OR alpaca[Tiab] OR alpacas[Tiab] OR camelid[Tiab] OR camelids[Tiab] OR guanaco[Tiab] OR guanacos[Tiab] OR chiroptera[Tiab] OR chiropteras[Tiab] OR bat[Tiab] OR bats[Tiab] OR fox[Tiab] OR foxes[Tiab] OR iguana[Tiab] OR iguanas[Tiab] OR xenopus laevis[Tiab] OR parakeet[Tiab] OR parakeets[Tiab] OR parrot[Tiab] OR parrots[Tiab] OR donkey[Tiab] OR donkeys[Tiab] OR mule[Tiab] OR mules[Tiab] OR zebra[Tiab] OR zebras[Tiab] OR shrew[Tiab] OR shrews[Tiab] OR bison[Tiab] OR bisons[Tiab] OR buffalo[Tiab] OR buffaloes[Tiab] OR deer[Tiab] OR deers[Tiab] OR bear[Tiab] OR bears[Tiab] OR panda[Tiab] OR pandas[Tiab] OR "wild hog"[Tiab] OR "wild boar"[Tiab] OR fitchew[Tiab] OR fitch[Tiab] OR beaver[Tiab] OR beavers[Tiab] OR jerboa[Tiab] OR jerboas[Tiab] OR capybara[Tiab] OR capybaras[Tiab]) NOT medline[subset])

Combined search: (#1 AND #2) AND (#3 OR #4) 140

**SCOPUS –** ***Search filters***	***Retrieved records***
**#1 Disease:**<br>((TITLE-ABS-KEY("Chagas disease") OR TITLE-ABS-KEY("American trypanosomiasis") OR TITLE-ABS-KEY("Trypanosoma cruzi") OR TITLE-ABS-KEY("Trypanosoma cruzus")))	*23.654*
**#2 Therapeutic target / Intervention:**<br>((TITLE-ABS-KEY("trypanothione reductase") OR TITLE-ABS-KEY("trypanothione reductase inhibitors")))	*495*

**Combined search:** #1 AND #2	*247*
Search limits: *in vitro, review, conference paper,* book chapter *[exclude]; animals [limit to]*	*175*

**WEB OF SCIENCE –** ***Search filters***	***Retrieved records***
**#1 Disease:**<br>TS=Chagas disease OR TS=American trypanosomiasis OR TS=Trypanosoma cruzi OR TS=Trypanosoma cruzus	*21.596*
**#2 Therapeutic target / Intervention:**<br>TS= trypanothione reductase OR TS= trypanothione reductase inhibitors	*866*
**#3 Animal filter:**<br>TS=mice OR TS=mouse OR TS=rat OR TS=rats OR TS=dog OR TS=dogs OR TS=rabbits OR TS=murine model OR TS=guinea pig OR TS=hamster OR TS=pig OR TS=animal model	*3.674.547*
**Combined search:** #1 AND #2 AND #3	*48*

Table S2. Analysis of methodological quality (reporting bias) of all studies included in the systematic review.

	Quality criteria / Studies included	Olmo et al. 2016	Fauro et al. 2013	Bazan et al. 2008	Rivarola et al. 2005	Lo Presti et al. 2004	Rivarola et al. 2001	Fournet et al. 2000	Baillet et al. 1996	Bazan et al. 2016	Rivarola et al. 1999	Strauss et al. 2013	Gobbi et al. 2010	Bustamente et al. 2010	Paglini-Oliva et al. 1998	Criteria completed (n)	Criteria completed (%)
	Title																
1	Accurate and concise description of the article content	Y	Y	Y	Y	Y	Y	Y	Y	Y	Y	Y	Y	Y	Y	15	100.0
	Abstract																
2	Background, research objectives, methods, principal findings and conclusions	Y	Y	Y	Y	Y	Y	Y	Y	Y	Y	Y	Y	Y	Y	15	100.0
	Introduction																
3	Sufficient scientific background	Y	Y	Y	Y	Y	Y	Y	Y	Y	Y	Y	Y	Y	Y	15	100.0
4	Explanation of the experimental approach	Y	Y	Y	Y	Y	Y	Y	Y	Y	Y	Y	Y	Y	Y	15	100.0
	Objectives																
5	Clear primary and secondary objectives	Y	Y	Y	Y	Y	Y	Y	Y	N	N	N	N	N	N	9	60.0

Y, criteria completed; N, criteria not completed.

Table S2 ***(continuation).*** Analysis of methodological quality (reporting bias) of all studies included in the systematic review.

	Quality criteria / Studies included	Olmo et al. 2016	Fauro et al. 2013	Bazan et al. 2008	Rivarola et al. 2005	Lo Presti et al. 2001	Rivarola et al. 2001	Fournet et al. 2000	Baillet et al. 1996	Bazan et al. 2016	Rivarola et al. 1999	Strauss et al. 2013	Gobbi et al. 2010	Bustamente et al. 2010	Paglini-Oliva et al. 1998	Criteria completed (n)	Criteria completed (%)
	Materials and Methods																
	Ethical statement																
6	Ethical permissions	Y	N	Y	Y	Y	N	N	N	Y	N	Y	Y	Y	N	9	60.0
	Study design																
7	The experiment blinded	N	N	N	N	N	N	N	N	N	N	N	N	N	N	0	0.0
8	Dosage of treatment	Y	Y	Y	Y	Y	Y	Y	Y	Y	Y	Y	Y	Y	Y	15	100.0
9	Route of administration	N	Y	Y	Y	Y	Y	Y	Y	Y	Y	Y	Y	Y	Y	14	93.3
10	Duration of treatment	Y	Y	Y	Y	Y	Y	Y	Y	Y	Y	Y	Y	Y	Y	15	100.0
11	Period of treatment administration	N	N	N	N	N	N	N	N	N	N	N	N	N	N	0	0.0
12	Site of treatment administration	N	Y	Y	Y	Y	Y	Y	Y	Y	Y	Y	Y	Y	Y	14	93.3
13	Rationale for dosage choice	N	N	N	N	N	Y	N	N	N	N	N	N	N	Y	2	13.3
14	Rational choice of administration rout	Y	N	N	N	N	N	N	N	N	N	N	N	N	N	1	6.7

Y, criteria completed; N, criteria not completed.

Table S2 *(continuation).* Analysis of methodological quality (reporting bias) of all studies included in the systematic review.

	Quality criteria / Studies included	Olmo et al. 2016	Fauro et al. 2013	Bazan et al. 2008	Rivarola et al. 2005	Lo Presti et al. 2001	Rivarola et al. 2001	Fournet et al. 2000	Baillet et al. 1996	Bazan et al. 2016	Rivarola et al. 1999	Strauss et al. 2013	Gobbi et al. 2010	Bustamente et al. 2010	Paglini-Oliva et al. 1998	Citeria completed (n)	Citeria completed (%)
	Experimental animals																
15	Strain of the animals	N	Y	Y	Y	Y	Y	N	N	Y	Y	Y	Y	Y	Y	12	80.0
16	Sex of the animals	Y	Y	N	Y	Y	Y	Y	Y	Y	Y	Y	Y	N	Y	12	80.0
17	Weight range of the animals	Y	Y	Y	Y	N	Y	N	Y	Y	Y	Y	Y	N	Y	11	73.3
18	Age of the animals	Y	N	N	N	N	N	Y	N	N	N	N	N	N	N	2	13.3
19	Previous procedures on the animals	N	N	N	N	N	N	N	N	N	N	N	N	N	N	0	0.0
	Housing and husbandry																
20	Housing of experimental animals (type of facility, cage or housing)	N	N	N	N	N	N	N	N	N	N	N	N	N	N	0	0.0
21	Experimental conditions (temperature, humidity and light cycle)	Y	N	N	N	N	N	N	N	N	N	N	N	N	N	1	6.7
22	Welfare-related assessments and interventions	N	N	N	N	N	N	N	N	N	N	N	N	N	N	0	0.0

Y, criteria completed; N, criteria not completed.

Table S2 *(continuation).* Analysis of methodological quality (reporting bias) of all studies included in the systematic review.

	Quality criteria / Studies included	Olmo et al. 2016	Fauro et al. 2013	Bazan et al. 2008	Rivarola et al. 2005	Lo Presti et al. 2001	Rivarola et al. 2001	Fournet et al. 2000	Baillet et al. 1996	Bazan et al. 2016	Rivarola et al. 1999	Strauss et al. 2013	Gobbi et al. 2010	Bustamente et al. 2010	Paglini-Oliva et al. 1998	Citeria completed (n)	Citeria completed (%)
	Sample size																
23	Report the total number of animals and animals in each group	Y	N	Y	Y	Y	Y	Y	N	Y	Y	Y	Y	Y	Y	12	80.0
24	Details of sample size calculation	N	N	N	N	N	N	N	N	N	N	N	N	N	N	0	0.0
	Animals allocation																
25	Full details of animals allocation (including randomization or matching)	N	N	N	N	N	N	Y	N	N	N	N	N	N	N	1	6.7
26	Order in which the animals and groups were treated and assessed	N	N	N	N	N	N	N	N	N	N	N	N	N	N	0	0.0
	Experimental outcomes																
27	Clear experimental outcomes assessed	Y	Y	Y	Y	Y	Y	Y	Y	Y	Y	Y	Y	Y	Y	15	100.0
	Statistical methods																
28	Statistical methods used in data analysis	N	Y	Y	Y	Y	Y	Y	N	Y	Y	Y	Y	Y	Y	12	80.0
29	Assess whether the data met the assumptions of the statistical approach	N	N	N	N	N	N	N	N	N	N	N	N	N	N	0	0.00

Y, criteria completed; N, criteria not completed.

Table S2 *(continuation).* Analysis of methodological quality (reporting bias) of all studies included in the systematic review.

	Quality criteria / Studies included	Olmo et al. 2016	Fauro et al. 2013	Bazan et al. 2008	Rivarola et al. 2005	Lo Presti et al. 2001	Rivarola et al. 2001	Fournet et al. 2000	Baillet et al. 1996	Bazan et al. 2016	Rivarola et al. 1999	Strauss et al. 2013	Gobbi et al. 2010	Bustamente et al. 2010	Paglini-Oliva et al. 1998	Citeria completed (n)	Citeria completed (%)
	Results																
	Baseline data																
30	Description of health status of animals before treatment	N	N	N	N	N	N	N	N	N	N	N	N	N	N	0	0.0
	Outcomes and estimation																
32	Animals or data not included in the analysis (explanation for exclusion)	N	N	N	N	N	N	N	N	N	N	N	N	N	N	0	0.0
33	Information on parasitemia	Y	Y	Y	Y	N	Y	Y	N	Y	Y	Y	Y	N	Y	11	73.3
34	Information regarding inflammation	N	Y	Y	N	Y	Y	N	N	N	Y	Y	Y	Y	Y	10	66.7
35	Information regarding animals' mortality	N	Y	Y	Y	Y	Y	Y	N	Y	Y	Y	N	Y	N	11	73.3

Y, criteria completed; N, criteria not completed.

Table S2 *(continuation)*. Analysis of methodological quality (reporting bias) of all studies included in the systematic review.

	Quality criteria / Studies included	Olmo et al. 2016	Fauro et al. 2013	Bazan et al. 2008	Rivarola et al. 2005	Lo Presti et al. 2001	Rivarola et al. 2001	Fournet et al. 2000	Baillet et al. 1996	Bazan et al. 2016	Rivarola et al. 1999	Strauss et al. 2013	Gobbi et al. 2010	Bustamente et al. 2010	Paglini-Oliva et al. 1998	Citeria completed (n)	Citeria completed (%)
	Discussion																
	Interpretation / scientific implications																
37	Interpretation of the results consider the objectives and hypotheses. current theory and relevant studies	Y	Y	Y	Y	Y	Y	Y	Y	Y	Y	Y	Y	Y	Y	15	100.0
38	Comments on the study limitations (sources of bias and limitations of the animal model)	N	N	N	N	N	N	N	N	N	N	N	N	N	N	0	0.0
	Generalizability/translation																
39	Comments on the relevance to human biology	Y	Y	Y	Y	Y	Y	Y	Y	Y	Y	Y	N	N	Y	13	86.7
	Funding																
40	List of funding sources	Y	Y	Y	Y	Y	Y	Y	Y	Y	Y	Y	Y	Y	Y	15	100.0
	**Criteria completed (n)**	19	20	24	21	20	22	20	15	20	20	21	19	17	20		**19.9 ± 2.1**
	**Criteria completed (%)**	50.0	52.6	63.2	55.3	52.6	58.0	52.6	39.5	52.6	52.6	55.3	50.0	44.7	52.6		**52.3 ± 5.6**

Y, criteria completed; N, criteria not completed.

Table S3. General characteristics of all studies investigating the effect of trypanothione reductase inhibitors on experimental Chagas disease.

Authors	Animal species	Lineage	Sex	Age	Weight	*T. cruzi* strain
Olmo et al. 2016	Mice	Balb/C	Female	6- 8 weeks	25 - 30 g	CL Brener (?)
Lo Presti et al. 2015	Mice	(-)	(-)	(-)	(-)	Tulahuen and SGO-Z12
Fauro et al. 2013	Mice	Swiss	Male and Female	(-)	30 ± 1g	Tulahuen
Bazan et al. 2008	Mice	Swiss	(-)	(-)	30 ± 1 g	Tulahuen
Rivarola et al. 2005	Mice	Swiss	Male	(-)	30 ± 1 g	SGO-Z12
Lo Presti et al. 2004	Mice	Swiss	Male	(-)	(-)	SGO-Z12
Rivarola et al. 2001	Mice	Swiss	Male		30 ± 1 g	Tulahuen
(-)Fournet et al. 2000	Mice	Balb/C	Male and Female	6 - 8 weeks	(-)	(-)
Baillet et al. 1996	Mice	Swiss	Female	(-)	20 - 25 g	(-)
Bazan et al. 2016	Mice	Swiss	Male and Female	(-)	30 ± 1 g	Tulahuen
Rivarola et al. 1999	Mice	Swiss	Male	(-)	30 ± 1 g	Tulahuen
Strauss et al. 2013	Mice	Swiss	Male and Female	(-)	30 ± 1 g	Tulahuen
Gobbi et al. 2010	Mice	Swiss	Male	(-)	30 ± 1 g	Tulahuen
Bustamante et al. 2007	Mice	Swiss	(-)	(-)	(-)	Tulahuen and SGO-Z12
Paglini-Oliva et al. 1998	Mice	Swiss	Male	(-)	30 ± 1 g	Tulahuen

(-) Data not reported or not investigated. (?) *T. cruzi* CL Brener strain was not reported for mice infection but was quoted in PCR analysis.

Table S3 (*continuation*). General characteristics of all included studies.

Authors	Treatment tested	Administration route	Dose	Treatment (days)
Olmo et al. 2016	Tetraamina-based drugs	(-)	5 mg/kg	5
Lo Presti et al. 2015	Thioridazine	Oral	80 mg/kg	Acute: 3 / Chronic: 12
Fauro et al. 2013	Clomipramine	Intraperitoneal	5 mg/kg	60
Bazan et al. 2008	Clomipramine	Intraperitoneal	5 mg/kg	30
Rivarola et al. 2005	Clomipramine	Intraperitoneal	5 and 40 mg/kg	30 and 60
Lo Presti et al. 2004	Thioridazine	Oral	80 mg/kg	3
Rivarola et al. 2001	Clomipramine	Intraperitoneal	5 and 40mg/kg	7 and 30
Fournet et al. 2000	Bisbenzylisoquinolone alkaloids	Oral	25 mg/kg	30
Baillet et al. 1996	Diphenylsulfide (2-amino diphenylsulfides) derivatives	Oral and intraperitoneal	40 mg/kg	4
Bazan et al. 2016	Clomipramine	Intraperitoneal	5 mg/kg	60
Rivarola et al. 1999	Thioridazine	Oral	80 mg/kg	3
Strauss et al. 2013	Clomipramine	Intraperitoneal	5 mg/kg	30
Gobbi et al. 2010	Clomipramine	Intraperitoneal and Oral	5 mg/kg;	90
Bustamante et al. 2007	Thioridazine	Oral	80 mg/kg	12 and 30
Paglini-Oliva et al. 1998	Thioridazine	Oral	4, 5, 6, 16 and 80 mg/kg	3

(-) Data not reported or not investigated.

Table S4. Parasitemia and mortality in control infected mice and those treated with trypanothione reductase inhibitors.

Study	Groups of treatment / animals per group (n)	Groups	Infection stage	Parasitemia (blood tryp./mL × 10^3)		Survival, n (%)
Olmo et al. 2016	Control infected (n=6)	Untreated	Acute phase	60.24	0.05	6 (100%)
	Compoud 2 (n=6)	5 mg/kg		38.27	0.05	6 (100%)
	Compoud 3 (n= 6)	5 mg/kg		40.00	0.04	6 (100%)
	Compoud 5 (n=6)	5 mg/kg		44.35	0.05	6 (100%)
Lo Presti et al. 2015	Control infected Tul (n=25)	Untreated	Acute phase	(-)	(-)	5 (20%)
	Infected treated Tul (n=60)	TZ (80 mg/kg)		(-)	(-)	48 (80%)
	Control infected SGO (n=42)	Untreated		(-)	(-)	26 (44%)
	Infected treated SGO (n=42)	TZ (80 mg/kg)		(-)	(-)	7 (17.5%)
Fauro et al. 2013	Control infected (n= 25)	Untreated	Chronic phase	(-)	(-)	4 (15%)
	Infected treated (n= 30)	CLO (5 mg/kg)		(-)	(-)	7 (22%)
Bazan et al. 2008	Control infected (n= 21)	Untreated	Acute phase	(-)	(-)	11 (48%)
	Infected treated (n= 37)	CLO (5 mg/kg)		101.13	13.98	33 (90%)
Lo Presti et al. 2004	Control infected (n= 42)	TZ (80 mg/kg)	Chronic phase	(-)	(-)	8 (20%)
	Infected treated (n= 42)					21 (50%)

(-) Data not reported or not investigated, TZ, Thioridazine; CLO, clomipramine; Ceph, cepharanthine; Daph, daphnoline; SGO, SGO-Z12 *T. cruzi* strain; TUL, Tulahuen *T. cruzi* strain. Parasitemia: mean and standard error (mean ± SE). Survival: absolute number and percentage, n (%).

Table S4 (*continuation*). Parasitemia and mortality in control infected mice and those treated with trypanothione reductase inhibitors.

Study	Groups of treatment / animals per group (n)	Groups	Infection stage	Parasitemia (blood tryp./mL × 10^3)		Survival, n (%)
Rivarola et al. 2005	Control infected (n= 40)<br>Clomipramine 5 (n= 40)<br>Clomipramine 40 (n= 40)	Untreated	Acute phase	455.20	52.04	12 (30%)
		CLO (5 mg/kg)		29.15	2.21	28 (70%)
		CLO (40 mg/kg)		69.66	1.62	24 (60%)
		Untreated	Chronic phase	0.00	0.00	(-)
		CLO (5 mg/kg)		0.00	0.00	(-)
		CLO (40 mg/kg)		0.00	0.00	(-)
Rivarola et al. 2001	Control infected (n= 20)<br>Clomipramine 5 (n= 40)<br>Clomipramine 40 (n= 40)	Untreated	Acute phase	93.19	19.79	14 (70%)
		CLO (5 mg/kg)		10.53	14.36	32 (80%)
		CLO (40 mg/kg)		7.34	14.36	36 (90%)
		Untreated	Chronic phase	0.00	0.00	16 (80%)
		CLO (5 mg/kg)		0.00	0.00	30 (75%)
		CLO (40 mg/kg)		0.00	0.00	28 (70%)

(-) Data not reported or not investigated, TZ, Thioridazine; CLO, clomipramine; Ceph, cepharanthine; Daph, daphnoline; SGO, SGO-Z12 *T. cruzi* strain; TUL, Tulahuen *T. cruzi* strain. Parasitemia: mean and standard error (mean ± SE). Survival: absolute number and percentage, n (%).

Table S4 (*continuation*). Parasitemia and mortality in control infected mice and those treated with trypanothione reductase inhibitors.

Study	Groups of treatment / animals per group (n)	Groups	Infection stage	Parasitemia (blood tryp./mL × 10^3)		Survival, n (%)
Fournet et al. 2000	Parasitological assays:					
	Control infected (n= 10-15)	Untreated	Acute phase	143.72	8.31	38 (85%)
	Cepharanthine (n= 10-15)	Ceph (25 mg/kg)		204.21	16.06	20 (57%)
	Daphnoline (n= 10-15)	Daph (25 mg/kg)		98.31	11.27	33 (74%)
	Mortality assays:					
	Control infected (n= 45)	Untreated	Chronic phase	0.54	0.02	30 (67%)
	Cepharanthine (n= 35)	Ceph (25 mg/kg)		3.36	0.19	20 (57%)
	Daphnoline (n= 45)	Daph (25 mg/kg)		0.27	0.013	25 (56%)
Bazan et al. 2016		Untreated	Acute phase	108.81	9.85	(-)
	Control infected (n= 10)	CLO (5 mg/kg)		(-)	(-)	(-)
	Clomipramine (n= 10)	Untreated	Chronic phase	(-)	(-)	6 (63%)
		CLO (5 mg/kg)		(-)	(-)	7 (67%)

(-) Data not reported or not investigated, TZ, Thioridazine; CLO, clomipramine; Ceph, cepharanthine; Daph, daphnoline; SGO, SGO-Z12 *T. cruzi* strain; TUL, Tulahuen *T. cruzi* strain. Parasitemia: mean and standard error (mean ± SE). Survival: absolute number and percentage, n (%).

Table S4 (*continuation*). Parasitemia and mortality in control infected mice and those treated with trypanothione reductase inhibitors.

Study	Groups of treatment / animals per group (n)	Groups	Infection stage	Parasitemia (blood tryp./mL × 10^3)		Survival, n (%)
Rivarola et al. 1999	Control infected (n= 20)<br>Thioridazine (n= 60)	Untreated	Acute phase	52.81	11.32	8 (40%)
		TZ (80 mg/kg)		12.49	2.06	54 (90%)
		Untreated	Chronic phase	0.00	0.00	16 (80%)
		TZ (80 mg/kg)		0.00	0.00	12 (20%)
Strauss et al. 2013	Control infected (n= 20)	Untreated	Acute phase	50.34	1.74	9 (45%)
	Clomipramine (n= 20)	CLO (5 mg/kg)		25.95	2.14	11 (55%)
Gobbi et al. 2010	Control infected (n= 20)	Untreated	Acute phase	38.07	6.40	(-)
	Clomipramine (n= 20)	CLO (5 mg/kg)		21.68	3.58	(-)
Bustamante et al. 2007	TUL infected (n= 25)	Untreated	Chronic phase	(-)	(-)	7 (30%)
	TUL inf. + Thioridazine (n= 25)	TZ (80 mg/kg)		(-)	(-)	22 (88%)
	SGO-Z12 infected (n= 20)	Untreated		(-)	(-)	8 (40%)
	SGO-Z12 inf. + Thioridazine (n= 20)	TZ (80 mg/kg)		(-)	(-)	18 (88%)
Paglini-Oliva et al. 1998	Control infected (n= 100)	Untreated	Acute phase	31.97	3.80	(-)
	Thioridazine (n= 25)	TZ (80 mg/kg)		31.20	1.80	(-)

(-) Data not reported or not investigated, TZ, Thioridazine; CLO, clomipramine; Ceph, cepharanthine; Daph, daphnoline; SGO, SGO-Z12 *T. cruzi* strain; TUL, Tulahuen *T. cruzi* strain. Parasitemia: mean and standard error (mean ± SE). Survival: absolute number and percentage, n (%).

Table S5. Parasitic load and cure rate in studies using polymerase chain reaction (PCR) or serology.

Study	Groups (mice)	Parasitic load (Parasite equivalent / ng DNA)	Cure, n (%)
Olmo *et al*. 2016*	Untreated (n=6)	(-)	0 (0%)
	Compound 2 (n=6)		2 (33.33%)
	Compound 3 (n=6)		3 (50%)
	Compound 5 (n=6)		0 (0%)
Fauro et al. 2013*	Untreated (n=25)	(-)	?
	CLO (5 mg/kg) (n=30)		
Strauss et al. 2013*	Untreated (n=20)	(-)	0 (0%)†
	CLO (5 mg/kg) (n=20)		0 (0%)†
Bazan et al. 2016*	Untreated (n=10)	90.47 ± 51.28	7 (75.87%)
	CLO (5 mg/kg) (n=10)	30.76 ± 7.69	8 (79.76%)
Fournet et al. 2000#	Untreated (n= 60)	(-)	10 (17%)
	Cepharanthine (n= 47)	(-)	24 (51%)
	Daphnoline (n= 55)	(-)	46 (84%)

*PCR, # Serology. (-) Not investigated. (?) Inconclusive analysis due to limited sample or absence of measures for each mice. †Information was reported in the results, but no graphical element was presented.

Table S6. Electrocardiographic data from control infected mice and those treated with trypanothione reductase inhibitors.

Authors	Days post-infection	Groups	Pulse (beats/min)	Axes (grade)	PQ Interval	QRS Interval
Rivarola et al. 2001	35	Untreated (n=20)	268.00 ± 5.53	57.00 ± 3.00	0.02-0.04	0.02-0.03
		CLO 5mg/kg (n=40)	260.00 ± 41.00	63.00 ± 2.84	0.02-0.04	0.02-0.04
		CLO 40mg/kg (n=40)	255.00 ± 4.43	60.00 ± 6.17	0.02-0.04	0.02-0.03
	75	Untreated (n=20)	263.00 ± 4.90	55.00 ± 3.95	0.02-0.03	0.02-0.04
		CLO 5mg/kg (n=40)	286.00 ± 3.48	65.00 ± 5.22	0.02-0.04	0.03-0.04
		CLO 40mg/kg (n=40)	282.00 ± 3.95	59.00 ± 4.27	0.02-0.05	0.03-0.04
	135	Untreated (n=20)	263.00 ± 6.48	55.00 ± 5.22	0.02-0.04	0.03-0.05
		CLO 5mg/kg (n=40)	260.00 ± 5.53	67.00 ± 4.74	0.02-0.02	0.02-0.03
		CLO 40mg/kg (n=40)	295.00 ± 4.43	57.00 ± 3.32	0.02-0.05	0.03-0.04
Bustamante et al. 2007	215	Untreated (Tul) (n=25)	570.00 ± 20.00	45.00 ± 10.00	0.027 ± 0.001	0.017 ± 0.001
		Tul + TZ 80mg/kg (n=25)	586.00 ± 10.00	31.00 ± 8.00	0.022 ± 0.001	0.015 ± 0.001
		Untreated (SGO-Z12) (n=19)	500.00 ± 15.00	50.00 ± 2.00	0.03 ± 0.002	0.035 ± 0.01
		SGO-Z12 + TZ 80mg/kg (n=20)	495.00 ± 15.00	41.00 ± 2.50	0.015 ± 0.001	0.015 ± 0.002
	350	Untreated (Tul) (n=25)	569.00 ± 29.00	38.00 ± 15.00	0.024 ± 0.001	0.015 ± 0.001
		Untreated (SGO-Z12) (n=19)	470.00 ± 12.00	48.00 ± 3.00	0.034 ± 0.01	0.035 ± 0.01
		Tul + TZ 80mg/kg (n=25)	586.00 ± 11.00	20.00 ± 6.00	0.022 ± 0.001	0.014 ± 0.001
		SGO-Z12 + TZ 80mg/kg (n=16)	515.00 ± 45.00	35.00 ± 5.50	0.02 ± 0.001	0.016 ± 0.001

Pulse and axes: mean and standard error (mean ± SE). PQ and QRS intervals: maximum and minimum.

Table S6 (*continuation*). Electrocardiographic data from infected untreated mice and those treated with trypanothione reductase inhibitors.

Authors	Days post-infection	Groups	Pulse (beats/min)	Axes (grade)	PQ Interval	QRS Interval
Rivarola et al. 1999	35	Untreated (n=10)	468.00 ± 12.10	51.00 ± 4.70	0.02-0.04	0.02-0.03
		TZ 80mg/kg (n=10)	498.00 ± 15.80	60.00 ± 5.10	0.02-0.04	0.02-0.03
	75	Untreated (n=10)	575.00 ± 4.80	44.00 ± 2.30	0.03-0.04	0.02-0.04
		TZ 80mg/kg (n=10)	519.00 ± 15.10	58.00 ± 4.90	0.02-0.04	0.02-0.04
	135	Untreated (n=10)	526.00 ± 16.90	55.00 ± 2.90	0.02-0.05	0.02-0.06
		TZ 80mg/kg (n=10)	503.00 ± 14.90	63.00 ± 3.80	0.02-0.04	0.02-0.04
Rivarola et al. 2005	55	Untreated (n=40)	501.80 ± 15.80	44.00 ± 6.00	0.015-0.03	0.02-0.04
		CLO 5mg/kg (n=40)	483.00 ± 54.70	32.00 ± 8.30	0.01-0.03	0.01-0.01
		CLO 40mg/kg (n=40)	464.00 ± 67.00	34.00 ± 27.00	0.01- 0.03	0.01-0.01
	90	Untreated (n=40)	509.70 ± 17.50	48.30 ± 8.40	0.02-0.04	0.02-0.04
		CLO 5mg/kg (n=40)	560.00 ± 54.70	36.00 ± 19.00	0.01-0.02	0.01-0.01
		CLO 40mg/kg (n=40)	560.00 ± 51.60	57.00 ± 8.20	0.01-0.02	0.01-0.01
	135	Untreated (n=40)	467.70 ± 12.00	38.20 ± 6.50	0.015-0.03	0.015-0.07
		CLO5mg/kg (n=40)	510.00 ± 22.00	57.00 ± 10.00	0.01-0.02	0.01-0.01
		CLO 40mg/kg (n=40)	517.00 ± 72.00	48.00 ± 15.20	0.01-0.02	0.01-0.01
Bazan et al. 2008	150	Untreated (n= 7)	668.00 ± 27.40	36.20 ± 4.9 0	N	N
		CLO 5mg/kg (n= 31)	622.00 ± 12.00	53.20 ± 4.12	N	N
Strauss et al. 2013	90	Untreated (n= 9)	491.25 ± 2.10	N	N	N
		CLO 5mg/kg (n=13)	500.50 ± 27.14	N	N	N

N, not investigated. Pulse and axes: mean and standard error (mean ± SE). PQ and QRS intervals: maximum and minimum.

Table S7. Electrocardiographic abnormalities and β-adrenergic receptors affinity and density in control and treated mice.

Authors	Days post-inf.	Groups	Abnormality, n (%)	β affinity (nM)	β density (mol/mg protein)
Rivarola et al. 2001	35	Untreated (n=20)	7 (35%)	5.63 ± 0.26	78.25 ± 1.67
		CLO 5mg/kg (n=40)	17 (42%)	7.46 ± 1.12	73.32 ± 4.73
		CLO 40mg/kg (n=40)	17 (43%)	4.83 ± 0.72	76.06 ± 4.18
	75	Untreated (n=20)	10 (50%)	6.86 ± 020	77.28 ± 091
		CLO 5mg/kg (n=40)	25 (62%)	0.900 ± 0.18	30.42 ± 1.59
		CLO 40mg/kg (n=40)	19 (48%)	2.93 ± 0.53	55.66 ± 4.12
	135	Untreated (n=20)	18 (90%)	11.21 ± 0.25	53.33 ± 0.71
		CLO 5mg/kg (n=40)	0 (0%)	4.56 ± 0.98	65.02 ± 4.88
		CLO 40mg/kg (n=40)	25 (63%)	4.43 ± 1.22	63.35 ± 6.01
Bustamante et al. 2007	215	Untreated (Tul) (n=20)	16 (65%)	N	N
		Tul + TZ 80mg/kg (n=40)	9 (36.7%)	N	N
		Untreated (SGO-Z12) (n=20)	12 (62%)	N	N
		SGO-Z12 + TZ 80mg/kg (n=40)	7 (33%)	N	N
	350	Untreated (Tul) (n=20)	17 (66.6%)	N	N
		Tul + TZ 80mg/kg (n=40)	11 (43.3%)	N	N
		Untreated (SGO-Z12) (n=20)	13 (63%)	N	N
		SGO-Z12 + TZ80mg/kg (n=40)	5 (33%)	N	N

N, not investigated. Affinity and density of β-adrenergic receptors: mean and standard error (mean ± SE).

Table S7 (*continuation*). Electrocardiographic abnormalities and β-adrenergic receptors affinity and density in control and treated mice.

Authors	Days post-inf.	Groups	Abnormality, n (%)	β affinity (nM)	β density (mol/mg protein)
Rivarola et al. 1999	35	Untreated (n=10)	1 (12.5%)	5.63 ± 0.27	78.25 ± 1.67
		TZ 80mg/kg (n=10)	2 (16%)	6.84 ± 0.36	76.85 ± 1.69
	75	Untreated (n=10)	7 (66%)	6.86 ± 0.21	77.28 ± 0.91
		TZ 80mg/kg (n=10)	4 (40%)	5.82 ± 0.32	74.37 ± 1.57
	135	Untreated (n=10)	6 (60%)	11.21 ± 0.26	53.33 ± 0.71
		TZ 80mg/kg (n=10)	4 (37%)	5.48 ± 0.21	72.34 ± 1.06
Rivarola et al. 2005	55	Untreated (n=40)	2 (18%)	5.71 ± 0.56	207.60 ± 8.10
		CLO 5mg/kg (n=40)	1 (11%)	9.05 ± 0.86	271.30 ± 10.10
		CLO 40mg/kg (n=40)	2 (17%)	6.06 ± 0.70	255.90 ± 10.00
	90	Untreated (n=40)	4 (36%)	6.28 ± 0.40	228.00 ± 6.02
		CLO 5mg/kg (n=40)	2 (20%)	8.10 ± 0.80	292.20 ± 11.20
		CLO 40mg/kg (n=40)	1 (10%)	5.32 ± 0.44	249.80 ± 6.60
	135	Untreated (n=40)	5 (55%)	7.32 ± 0.19	184.10 ± 2.10
		CLO 5mg/kg (n=40)	2 (18%)	7.74 ± 0.94	302.40 ± 13.6
		CLO40mg/kg (n=40)	2 (16%)	4.90 ± 0.48	230.60 ± 6.98

N, not investigated. Affinity and density of β-adrenergic receptors: mean and standard error (mean ± SE).

Table S7 (*continuation*). Electrocardiographic abnormalities and β-adrenergic receptors affinity and density in control and treated mice.

Authors	Days post-inf.	Groups	Abnormality, n (%)	β affinity (nM)	β density (mol/mg protein)
Bazan et al. 2008	150	Untreated (n= 7)	N	11.20 ± 0.26	53.30 ± 0.71
		CLO 5mg/kg	N	6.27 ± 0.23	77.20 ± 1.08
Bazan et al. 2016	90	Untreated (n=10)	4 (36%)	N	N
		CLO 5mg/kg (n=10)	3 (33%)	N	N
	180	Untreated (n=10)	5 (50%)	N	N
		CLO5mg/kg (n=10)	6 (64%)	N	N
	270	Untreated (n=10)	6 (57%)	N	N
		CLO 5mg/kg (n=10)	2 (20%)	N	N

N, not investigated. N, not investigated. Affinity and density of β-adrenergic receptors: mean and standard error (mean ± SE).

Table S8. Descriptive results obtained in infected mice treated with trypanothione reductase inhibitors compared to infected untreated mice.

Authors	Treatment	Immunological, histopathological and biochemical findings	Electrocardiographic findings
Olmo et al. 2016	Tetraamine-based compounds	Compound 3 reduced uric acid, urea and CK-MB. LDH levels (30% - 40%) in treated mice	N
Lo Presti et al. 2015	Thioridazine	Reduced necrosis, fibrosis and inflammatory infiltrate in treated mice	Prevented cardiomyopathy progression and reduced electrocardiographic abnormalities (cardiac blockades)
Fauro et al. 2013	Clomipramine	Reduced myocarditis, fibrosis, anti-*T. cruzi* antibodies serum levels and parasite load	N
Bazan et al. 2008	Clomipramine	Reduced heart inflammatory infiltrate and necrosis	Reduced cardiomyopathy progression and electrocardiographic abnormalities
Rivarola et al. 2005	Clomipramine	Similar anti-cruzipain IgG serum levels and reduced inflammatory infiltrate	Reduced electrocardiographic abnormalities (pulse, axes, PQ and QRS intervals)
Rivarola et al. 2005	Clomipramine	Similar anti-cruzipain IgG serum levels and reduced inflammatory infiltrate	Reduced electrocardiographic abnormalities (pulse, axes, PQ and QRS intervals)

*Benznidazole was used as a reference antitrypanosomal drug. N, not investigated.

Table S8 *(continuation).* Descriptive results obtained in infected mice treated with trypanothione reductase inhibitors compared to infected untreated mice.

Authors	Treatment	Immunological, histopathological and biochemical findings	Electrocardiographic findings
Lo Presti et al. 2004	Thioridazine	Similar anti-cruzipain IgG serum levels and reduced inflammatory infiltrate	Reduced β-receptors affinity and higher β-receptors density
Rivarola et al. 2001	Clomipramine	Reduced anti-cruzipain IgG antibody serum levels in treated groups	Reduced receptor density, affinity and electrocardiographic abnormalities (pulse, axes, PQ and QRS intervals)
Fournet et al. 2000	Bisbenzylisoquin-olone alkaloids	N	N
Baillet et al. 1996	Diphenylsulfide derivatives	N	N
Bazan et al. 2016	Clomipramine	Similar anti-*T. cuzi* antigens serum levels. Reduced parasitic DNA in blood samples	Attenuation of intra-ventricular block and arrhythmias
Rivarola et al. 1999	Thioridazine	Similar anti-cruzipain IgG serum levels, reduced inflammatory infiltrate and absence of amastigote nests	Attenuation of intra-ventricular block and arrhythmias. Reduced receptor affinity and higher density

N, not investigated.

Table S8 *(continuation).* Descriptive results obtained in infected mice treated with trypanothione reductase inhibitors compared to infected untreated mice.

Authors	Treatment	Immunological, histopathological and biochemical findings	Electrocardiographic findings
Strauss et al. 2013	Clomipramine	Absence of necrosis, similar fibrosis and inflammatory infiltrate. No evidences of liver and kidney toxicity.	No prevented prolonged PR interval (atrioventricular block)
Gobbi et al. 2010	Clomipramine	Similar anti-cruzipain IgG serum levels and reduced myocardial damage	Reduced β-receptor affinity and higher density. No electrocardiographic abnormalities
Bustamante et al. 2007	Thioridazine	Reduced heart necrosis, fibrosis and inflammatory infiltrate	Reduced β-receptor density, affinity, and electrocardiographic abnormalities (pulse, axes, PQ and QRS intervals)
Paglini-oliva et al. 1998	Thioridazine	Reduced inflammatory infiltrates and no amastigote nests	Prevented cardiomyopathy progression

N, not investigated.
